# IL-1β/EPAS1-Associated Ferroptotic Stress Impairs Skeletal Stem/Progenitor Cell Function in Inflammation-Associated Fracture Nonunion

**DOI:** 10.3390/cimb48060606

**Published:** 2026-06-09

**Authors:** Ruoyu Wang, Jie Li, Yu Zhai, Qin Song, Pengyu Xia, Bowen Jiang, Minghang Chen, Minghan Liu, Changqing Li

**Affiliations:** 1 Chongqing Municipal Health Commission Key Laboratory of Precise Orthopedics, Army Medical University (Third Military Medical University), Chongqing 400038, China; wangruoyu2000@tmmu.edu.cn (R.W.); lblijie@tmmu.edu.cn (J.L.);; 2 Chongqing Municipal Health Commission Key Laboratory of Musculoskeletal Regeneration and Translational Medicine, Orthopedic Research Laboratory of Chongqing Medical University, Department of Orthopedic Surgery, The First Affiliated Hospital of Chongqing Medical University, Chongqing 400016, China; 3 Department of Orthopedics, The Second Affiliated Hospital (Xinqiao Hospital), Army Medical University (Third Military Medical University), Chongqing 400038, China

**Keywords:** fracture nonunion, ferroptosis, IL-1β, EPAS1, single-cell RNA sequencing, skeletal stem/progenitor cells, Mendelian randomization

## Abstract

Atrophic fracture nonunion is a clinically challenging form of failed bone repair, particularly under inflammatory conditions, but the cell-intrinsic programs that impair the function of skeletal stem/progenitor cells (SSPCs) remain incompletely defined. Here, we integrated public and in-house single-cell RNA sequencing datasets from mouse periosteum, normal fracture healing, and inflammation-associated fracture nonunion models to characterize stromal cell fate changes. Trajectory inference, transcription factor network analysis, and intercellular communication modeling were combined with in vitro and in vivo validation experiments. SSPCs in the nonunion microenvironment were arrested in an undifferentiated state and acquired a pro-inflammatory and pro-ferroptotic phenotype, with enrichment of ferroptosis-related genes including *Acsl4*. Computational analyses nominated IL-1β as a candidate upstream inflammatory signal, with neutrophils representing a potential source, and linked this signal to NF-κB activation and increased *Epas1* activity in SSPCs. In primary SSPCs, IL-1β induced lipid peroxidation, intracellular ferrous iron accumulation, ferroptosis-related protein expression, and impaired osteochondrogenic differentiation. Ferroptosis inhibitor treatment further attenuated IL-1β-induced ferroptosis-related protein changes, supporting pathway specificity. Pharmacological inhibition of EPAS1 with PT2385 attenuated IL-1β-induced ferroptotic *stress* and restored SSPC differentiation in vitro, while also improving IL-1β-impaired fracture repair in vivo. Mendelian randomization analysis provided additional genetic evidence supporting potential links among IL-1β, EPAS1, and human nonunion risk. Together, these findings suggest that an IL-1β/EPAS1-associated ferroptotic program contributes to SSPC dysfunction during inflammation-associated fracture nonunion and may represent a potential targetable mechanism for improving impaired bone repair.

## 1. Introduction

Most bone fractures heal through a coordinated regenerative process, yet approximately 5–10% progress to delayed union or nonunion [1,2]. Atrophic nonunion represents a biologically impaired form of failed repair, characterized by limited callus formation, poor vascularization, and fibrotic tissue accumulation at the fracture site [3,4,5]. Although mechanical instability is an important cause of some nonunions, the atrophic phenotype indicates that defective local tissue regeneration is a central component of disease pathogenesis [3,5]. Current surgical and biological interventions remain variably effective, highlighting the need to define the cellular and molecular mechanisms underlying this regenerative failure [6,7].

Systemic inflammation is closely associated with impaired fracture healing [8]. Clinical conditions such as rheumatoid arthritis, diabetes, and aging are linked to delayed bone repair and increased nonunion risk [9,10,11]. Consistently, TNFα-transgenic and serum-transfer-induced inflammatory arthritis models show defective bone regeneration and atrophic nonunion-like features [12,13,14]. At the molecular level, persistent inflammation activates canonical NF-κB signaling and increases inflammatory mediators, including interleukin-1β (IL-1β) and TNF-α [15]. Although these inflammatory cues can affect angiogenesis and other repair-related processes [16], the effects of cytokine-targeted interventions on fracture healing remain inconsistent [17,18]. Thus, the cell-intrinsic mechanisms by which inflammatory cytokines, particularly IL-1β, impair skeletal repair remain insufficiently defined.

Skeletal stem/progenitor cells (SSPCs), especially periosteum-derived progenitors, are essential for osteochondral repair during fracture healing [19,20]. Recent single-cell and single-nucleus transcriptomic studies have shown that SSPCs pass through an injury-induced fibrogenic cell (IIFC) state before differentiating toward chondrogenic and osteogenic lineages [21]. During normal repair, this transient intermediate state appears to support tissue regeneration. Under pathological inflammatory conditions, however, pro-inflammatory stromal and immune states may persist, contributing to fibrotic tissue accumulation and failed bone formation [21,22]. These observations suggest that inflammation-associated nonunion may involve a pathological shift in SSPC fate rather than only a reduction in progenitor abundance.

Ferroptosis is an iron-dependent form of regulated cell death driven by lipid peroxidation and is closely linked to oxidative stress and inflammatory signaling [23,24]. It has been implicated in inflammatory and degenerative conditions, including ischemia–reperfusion injury and osteoarthritis [25,26,27,28]. Because the nonunion microenvironment contains persistent inflammatory and oxidative stress signals [29,30], ferroptosis may provide a mechanism connecting inflammatory stimulation to SSPC dysfunction. However, whether SSPCs acquire a ferroptosis-associated state during inflammation-associated fracture nonunion, and which upstream signals regulate this process, remains unclear.

EPAS1, which encodes hypoxia-inducible factor-2α (HIF-2α), may link inflammatory signaling to skeletal lineage remodeling and iron-dependent lipid peroxidation. In chondrocytes, IL-1β induces HIF-2α in part through NF-κB signaling, and HIF-2α promotes catabolic and hypertrophic programs involving MMP13, COL10A1, and ADAMTS4 [31,32]. IL-1β has also been reported to trigger chondrocyte ferroptosis by disrupting iron homeostasis and the SLC7A11/GPX4 antioxidant axis [33]. Importantly, HIF-2α can increase chondrocyte susceptibility to ferroptosis, and HIF-2α activation has been linked to increased cellular iron, lipid peroxidation, and ferroptosis-related regulators such as HILPDA and PLIN2 [34,35]. These findings suggest that EPAS1/HIF-2α may provide a mechanistic bridge between IL-1β-driven inflammation, altered iron-dependent lipid peroxidation, and ferroptosis-associated SSPC dysfunction. However, whether IL-1β activates an EPAS1-associated ferroptotic program in SSPCs during inflammation-associated fracture nonunion remains unclear.

In this study, we integrated public and in-house single-cell RNA sequencing datasets to examine stromal cell remodeling during normal fracture healing and inflammation-associated fracture nonunion. We combined trajectory inference, transcription factor network analysis, and intercellular communication modeling to identify candidate inflammatory signals and downstream regulatory programs associated with SSPC dysfunction. We then performed in vitro and in vivo experiments to assess whether IL-1β induces an EPAS1-associated ferroptotic program in SSPCs and whether pharmacological EPAS1 inhibition can attenuate ferroptotic stress, impaired differentiation, and defective bone repair. Finally, Mendelian randomization was used to explore the potential relevance of IL-1β and EPAS1 to human nonunion risk. These analyses support a model in which inflammatory IL-1β signaling is associated with diversion of SSPCs from osteochondrogenic repair toward an EPAS1-related ferroptotic state.

## 2. Materials and Methods

### 2.1. Data Sources and scRNA-Seq Processing

Single-cell RNA sequencing (scRNA-seq) data from mouse fracture nonunion (NonU) and normal fracture healing (Frac) models were obtained from the publicly available GEO dataset GSE242836. Callus tissues in this dataset were collected at 4 and 7 days post-fracture. In-house scRNA-seq data from uninjured periosteal tissue were used as the periosteum (PO) baseline control, and will be available upon reasonable request.

Raw scRNA-seq data were processed using Seurat v5.2.0 in R v4.4.2 [36]. Cells with fewer than 300 or more than 7200 detected genes, or with more than 5% mitochondrial gene content, were excluded. Potential doublets were identified and removed using DoubletFinder v2.0.3. After quality control, 47,549 cells were retained for downstream analysis. Batch effects across samples were corrected using Harmony v1.2.3 [37]. Principal component analysis was performed on highly variable genes, and unsupervised clustering was conducted using the shared nearest-neighbor graph-based method implemented in Seurat. Cell clusters were visualized using uniform manifold approximation and projection (UMAP). Cell types were annotated according to canonical marker genes and a previously reported fracture cell atlas [21,22].

### 2.2. Functional Annotation and Pathway Activity Analysis

Ferroptosis driver and suppressor gene sets were obtained from FerroDB v2 (http://www.zhounan.org/ferrdb/current/, accessed on 1 October 2025) [38]. Module scores for ferroptosis, inflammatory signatures, and lineage-associated gene programs were calculated using the AddModuleScore function in Seurat. Differentially expressed genes between NonU and Frac conditions were identified using FindMarkers, with adjusted *p* < 0.05 and |log2FC| > 1 used as the significance threshold. Gene Ontology (GO) and Kyoto Encyclopedia of Genes and Genomes (KEGG) enrichment analyses were performed using clusterProfiler v4.10.0 [39]. Pathway activity, including NF-κB pathway activity, was inferred using PROGENy v1.20.0 [40].

### 2.3. Trajectory, Gene Regulatory Network, and Cell–Cell Communication Analyses

Stromal lineage trajectories were inferred using Slingshot v2.8.0 on the UMAP embedding of the stromal cell subset, with the SSPC cluster assigned as the starting population [41]. Pseudotime-dependent changes in lineage markers, ferroptosis-related genes, and transcription factor-associated genes were examined along the inferred trajectories. Gene regulatory networks were reconstructed using SCENIC v1.3.1 to estimate regulon activity in SSPCs and identify transcription factors associated with the pro-ferroptotic SSPC state [42]. Intercellular communication was modeled using NicheNet v1.0.1, with SSPCs defined as the receiver population and major immune, stromal, and vascular cell types as sender populations [43]. Ligand activity and predicted ligand–target relationships were used to identify candidate upstream inflammatory signals associated with SSPC transcriptional changes, particularly those linked to the NF-κB/EPAS1-associated ferroptosis program.

### 2.4. Mendelian Randomization (MR) Analysis

This study was conducted and reported in accordance with the STROBE-MR guidelines (Strengthening the Reporting of Observational Studies in Epidemiology using Mendelian Randomization) [44]. The MR analysis was performed based on three core assumptions: (1) the genetic variants are associated with the exposure; (2) the variants are not associated with any confounders; and (3) the variants affect the outcome only through the exposure. Then, the analysis was performed to assess the potential causal relationships between the expression of ferroptosis-related TFs and the risk of bone nonunion, using the TwoSampleMR (v0.6.22) package in R software (version 4.4.1). Summary statistics for the genetic instruments associated with the TFs (*EPAS1, CEBPB, JUN, JUND*) and inflammatory exposures (blood neutrophil count: ieu-b-34, IL-1β protein level: prot-c-3037_62_1, and C-reactive protein level: ieu-b-35) were obtained from the OPEN GWAS database. Summary statistics for bone nonunion (finngen_R10_M13_BONE_CONTINUITY) were obtained from the FinnGen database. To ensure the robustness of our causal inferences, we strictly controlled for potential biases. Regarding population stratification and sample overlap, exposure and outcome analyses were restricted to European ancestry for consistent LD structures, with no known sample overlap between exposure and outcome datasets to minimize weak instrument bias and ensure two-sample independence.

Single-nucleotide polymorphisms (SNPs) significantly associated with the exposure (*p* < 5 × 10^−6^) were selected as potential instrumental variables. To ensure independence, these SNPs were clumped based on a linkage disequilibrium (LD) threshold of r^2^ < 0.001 within a 10,000 kb window. The primary causal estimate was calculated using the inverse-variance weighted (IVW) method. Several sensitivity analyses were performed to assess the robustness of the findings, including the weighted median method, MR-Egger regression, simple mode, and weighted mode methods. Pleiotropy was assessed using the MR-Egger intercept test, and heterogeneity was evaluated using Cochran’s Q statistic. A leave-one-out analysis was conducted to identify any single SNP driving the causal estimate. The study protocol was not pre-registered, as this work represents an exploratory analysis based on publicly available summary statistics. Reverse MR was performed using bone nonunion-associated SNPs as instruments to assess potential reverse associations with IL-1β and *EPAS1*.

### 2.5. Animals and Ethics Statement

All animal procedures were approved by the Animal Care Committee of Army Medical University (Third Military Medical University) (IACUC No. AMUWEC20255420). Animal experiments were conducted in accordance with institutional guidelines and the ARRIVE reporting recommendations. C57BL/6 male mice aged 8 weeks and weighing 20–25 g were used. Mice were purchased from Ensiweier Biotechnology Co., Ltd. (Chongqing, China) and housed in a specific-pathogen-free facility under a 12 h light/dark cycle with ad libitum access to food and water.

Mice were randomly assigned to four groups: vehicle control, IL-1β, IL-1β plus PT2385, and PT2385 alone, with six mice per group. Each mouse was considered one biological replicate for in vivo analyses. Healthy mice without visible limb abnormalities were included. Mice with perioperative death, severe infection, fixation failure, or fracture patterns inconsistent with the predefined model were excluded from analysis. Imaging and histological quantification were performed by investigators blinded to treatment allocation.

### 2.6. Open Mouse Femoral Fracture Model and In Vivo Treatment

A stabilized open mouse femoral fracture model was used to evaluate the effects of IL-1β and EPAS1 inhibition on fracture repair. Mice were anesthetized with isoflurane inhalation anesthesia. After shaving and sterile preparation, a lateral incision was made over the femur to expose the mid-diaphyseal region. A transverse femoral fracture was generated using a standardized open osteotomy procedure under direct visualization. The femur was stabilized with a 0.5 mm intramedullary stainless-steel pin, and the wound was closed in layers. Postoperative analgesia was provided according to institutional animal care guidelines.

To establish an inflammatory fracture-repair condition, recombinant mouse IL-1β was locally administered around the fracture site. Mice in the IL-1β group received local peri-fracture injection of IL-1β at 100 ng in 20 μL sterile PBS per mouse on postoperative days 0, 2, 4, and 6. Mice in the IL-1β plus PT2385 group received the same IL-1β treatment together with PT2385 administration. PT2385 (MedChemExpress, Monmouth Junction, NJ, USA; #HY-12867) was dissolved in 10% DMSO and 90% corn oil and administered by oral gavage at 50 mg/kg body weight once daily. Mice in the corresponding control groups received equal volumes of vehicle. Mice were euthanized at 28 days post-fracture, and femoral callus tissues were collected for micro-CT reconstruction and histological analysis. The detailed quantification procedures are described in Section 2.15.

### 2.7. Isolation and Culture of Primary SSPCs

To isolate SSPCs, callus tissue was collected at 7 days post-fracture. The tissue was flushed from femurs, and red blood cells were lysed. The remaining cells were stained with an APC/Cyanine7-conjugated lineage antibody cocktail, including antibodies against CD31 (BioLegend, San Diego, CA, USA, 102439), CD45 (BioLegend, San Diego, CA, USA, 157203), and Ter-119 (BioLegend, San Diego, CA, USA, 116223), and a PE-conjugated anti-CD26 (DPP4) antibody (BioLegend, 137804). SSPCs were isolated as the Lin^−^/DPP4^+^ population using a fluorescence-activated cell sorter. Sorted cells were cultured in standard medium for subsequent experiments. For all in vitro cell culture experiments, the experimental unit was defined as an individual culture well. For all in vitro cell culture experiments, the experimental unit was defined as an individual culture well. All experiments were performed with three independent biological replicates, and each biological replicate included three technical replicates.

### 2.8. In Vitro Treatments and Tri-Lineage Differentiation

For short-term mechanistic experiments, primary SSPCs were treated with vehicle, erastin (10 μM), or recombinant mouse IL-1β (10 ng/mL) for 48 h [25,27]. Erastin was used as a positive control for ferroptosis induction.

For ferroptosis inhibitor-based pathway validation, SSPCs were assigned to four groups: vehicle control, IL-1β, Fer-1, and IL-1β plus Fer-1. Cells were treated with recombinant mouse IL-1β (10 ng/mL), Ferrostatin-1 (Fer-1; MedChemExpress, Monmouth Junction, NJ, USA; #HY-100579; 1 μM), or their combination for 24 h. Cells were then collected for Western blot analysis of ferroptosis-related proteins and, where indicated, FerroOrange staining to assess intracellular ferrous iron accumulation.

To evaluate the effects of ferroptotic stress on differentiation potential, SSPCs were treated with IL-1β (10 ng/mL) or erastin (10 μM) only during the first 72 h of differentiation culture. After this induction period, cells were washed with PBS and cultured in fresh drug-free differentiation medium for the remainder of the 21-day differentiation period. The medium was refreshed every 3 days. Osteogenesis was assessed by Alizarin Red S staining, adipogenesis by Oil Red O staining, and chondrogenesis by Alcian Blue staining.

For the EPAS1 inhibition rescue experiment, SSPCs were treated with IL-1β in the presence or absence of PT2385. PT2385 (MedChemExpress, Monmouth Junction, NJ, USA; #HY-12867) was dissolved in DMSO to prepare a stock solution and diluted in culture medium immediately before use. The final concentration of DMSO was kept below 0.1% in all groups. Cells were assigned to four groups: vehicle control, IL-1β, IL-1β plus PT2385, and PT2385 alone. PT2385 was used at 10 μM. In the rescue condition, SSPCs were pretreated with PT2385 for 1 h before IL-1β stimulation and then co-treated with PT2385 and IL-1β for the indicated experimental period. For short-term assays, cells were treated for 48 h and collected for Western blot analysis, BODIPY 581/591 C11 staining, FerroOrange staining, and qRT-PCR. For differentiation assays, cells were exposed to the indicated treatments during the first 72 h of osteogenic or chondrogenic induction and then maintained in fresh drug-free differentiation medium until day 21.

### 2.9. Lipid Peroxidation Staining

To assess cellular lipid peroxidation, SSPCs cultured on coverslips were stained using the BODIPY 581/591 C11 probe (Thermo Fisher Scientific, Waltham, MA, USA, #D3861). Following experimental treatments, cells were washed three times with sterile PBS. The stock solution was freshly diluted to a 10 µM working concentration in serum-free medium. Cells were then incubated with the 10 µM working solution for 30 min at 37 °C, protected from light. After incubation, the working solution was removed, and cells were washed three times with sterile PBS. Images were immediately acquired using a Zeiss LSM 880 laser confocal microscope (Zeiss, Oberkochen, Germany). As BODIPY 581/591 C11 is a ratiometric probe, lipid peroxidation levels were quantified by calculating the fluorescence intensity ratio of the oxidized form (green, ~510 nm emission) to the reduced form (red, ~591 nm emission) using ImageJ software (version 1.54r, National Institutes of Health, Bethesda, MD, USA). Randomly selected fields were quantified for each sample, and the mean value was used for statistical analysis. Images from different treatment groups within the same experiment were acquired using identical microscope settings.

### 2.10. Intracellular Ferrous Iron Detection

To assess the intracellular ferrous iron (Fe^2+^) levels, the FerroOrange probe (Dojindo, Kumamoto, Japan, #F374) was utilized. Primary SSPCs were seeded in confocal dishes and treated as described above. Following treatment, cells were washed three times with serum-free medium to remove extracellular iron. The cells were then incubated with 1 μM FerroOrange working solution in serum-free medium for 30 min at 37 °C. Images were acquired using a Zeiss LSM 880 laser confocal microscope (Zeiss, Oberkochen, Germany). The fluorescence intensity was quantified using ImageJ software. Background-subtracted mean fluorescence intensity was calculated from randomly selected fields for each sample. All images within the same experiment were acquired using identical laser and exposure settings.

### 2.11. Western Blot Analysis

Total protein was extracted from treated SSPCs using RIPA lysis buffer (Beyotime, Shanghai, China) supplemented with protease and phosphatase inhibitor cocktails (Roche, Basel, Switzerland). Protein concentrations were determined using a BCA Protein Assay Kit (Thermo Fisher Scientific, Waltham, MA, USA). Equal amounts of protein lysates (20–30 μg) were separated by SDS-PAGE and transferred onto 0.45 μm polyvinylidene difluoride membranes (Merck Millipore, Billerica, MA, USA). Membranes were blocked with 5% non-fat milk in Tris-buffered saline containing 0.1% Tween-20 for 1 h at room temperature and incubated overnight at 4 °C with primary antibodies against ACSL4 (Proteintech Group, Rosemont, IL, USA, #22401-1-AP), COX2 (Proteintech Group, Rosemont, IL, USA, #27308-1-AP), NF-κB p65 (Proteintech Group, Rosemont, IL, USA, #80979-1-RR), EPAS1 (Thermo Fisher Scientific, #PA1-16510), 4-HNE (Abcam, Cambridge, UK, ab48506), β-actin (Proteintech Group, Rosemont, IL, USA, #66009-1-Ig), and GAPDH (Proteintech Group, Rosemont, IL, USA, #10494-1-AP). After washing, membranes were incubated with horseradish peroxidase-conjugated secondary antibodies (Abcam, Cambridge, UK, Cat# ab6789) for 1 h at room temperature. Protein bands were visualized using an enhanced chemiluminescence detection system (Thermo Fisher Scientific, Waltham, MA, USA). Band intensities were quantified using ImageJ. For the initial IL-1β/erastin validation experiments, protein expression was normalized to β-actin. For the EPAS1 inhibition rescue experiments, protein expression was normalized to GAPDH.

### 2.12. Colony Formation Assay

To assess self-renewal capacity, SSPCs were seeded into 6-well plates at a density of 1000 cells per well. The cells were cultured in standard medium containing Vehicle, IL-1β (10 ng/mL), or erastin (10 µM) for 10–14 days to allow colony formation. The medium was refreshed every 3 days. At the end of the culture period, cells were washed with PBS, fixed with 4% paraformaldehyde (PFA) for 15 min, and stained with 0.1% Crystal Violet solution (Beyotime, Shanghai, China) for 15 min. Excess dye was washed away with water, and the plates were air-dried. Colonies containing more than 50 cells were counted and imaged. Colony numbers were quantified per well, and each well was treated as an experimental unit.

### 2.13. Cell Viability Assay

Cell viability and proliferation were evaluated using the Cell Counting Kit-8 (CCK-8; Dojindo, Kumamoto, Japan) according to the manufacturer’s instructions. SSPCs were seeded into 96-well plates at a density of 1000 cells per well and treated with the indicated conditions. At the specified time point, 10 µL of CCK-8 reagent was added to each well. The plates were incubated at 37 °C for 2 h, protected from light. The absorbance (optical density) was measured at 450 nm using a microplate reader. Blank wells containing medium and CCK-8 reagent without cells were used for background correction. Absorbance values were normalized to the corresponding control group, where indicated.

### 2.14. RNA Isolation and Quantitative Real-Time PCR (qRT-PCR)

Total RNA was extracted from SSPCs using a Total RNA Extraction Kit (Solarbio, Beijing, China) following the manufacturer’s protocol. Genomic DNA elimination and cDNA synthesis were performed using the RevertAid First Strand cDNA Synthesis Kit (Thermo Fisher Scientific, USA). Quantitative real-time PCR (qRT-PCR) was conducted using SYBR Green Master Mix on a CFX96 Real-Time PCR Detection System (Bio-Rad Laboratories, Hercules, CA, USA). The amplification program consisted of an initial denaturation at 95 °C for 30 s, followed by 40 cycles of 95 °C for 5 s and 60 °C for 30 s. Relative gene expression was calculated using the 2^−∆∆Ct^ method, normalized to the housekeeping gene *Gapdh*.

### 2.15. Micro-CT and Histological Analysis

Femurs were harvested at 28 days post-fracture and fixed in 4% paraformaldehyde for 48 h. After fixation, specimens were transferred to PBS (pH 7.4) and stored at 4 °C before scanning.

For micro-computed tomography (micro-CT) analysis, fractured femurs were scanned using a VivaCT40 micro-CT system (Scanco Medical, Brüttisellen, Switzerland). The scanning parameters were kept constant across all samples: tube voltage, 70 kV; tube current, 114 μA; integration time, 300 ms; and isotropic voxel size, 15 μm. Three-dimensional reconstruction was performed using the manufacturer’s built-in software. The region of interest was defined as the fracture callus region centered on the fracture line and extending an equal distance proximally and distally. A fixed threshold was applied consistently across all samples to segment mineralized tissue. Bone morphometric parameters, including bone volume (BV) and bone volume fraction (BV/TV), were calculated using the scanner-associated analysis software. The same region-of-interest definition and segmentation threshold were applied to all samples.

After micro-CT scanning, femoral specimens were decalcified in 10% EDTA at 4 °C until no resistance was detected by needle testing. The specimens were then dehydrated, cleared, embedded in paraffin, and sectioned longitudinally at a thickness of 10 μm. Sections were deparaffinized, rehydrated, and subjected to Safranin O/Fast Green staining according to standard protocols to evaluate callus structure, cartilage matrix, and newly formed bone tissue. Histological images were acquired using a light microscope. Bone area and callus area were quantified using ImageJ software. For each sample, sections with comparable anatomical orientation around the fracture site were selected for quantification. All micro-CT and histological analyses were performed by investigators blinded to group allocation.

### 2.16. Statistical Analysis

Sample sizes were determined based on previous experience with similar experimental designs and standard practice in the field. For in vitro experiments, cells were randomly allocated to treatment groups, and sample processing order was randomized when applicable. Unless otherwise stated, each independent culture well was considered one experimental unit for in vitro assays. For in vivo experiments, mice were randomly assigned to treatment groups. Each mouse was considered one biological replicate for in vivo analyses. Imaging, histological quantification, and data analysis were performed by investigators blinded to group allocation.

All statistical analyses and data visualizations were performed using R v4.4.2 and GraphPad Prism v8.0. Data are presented as mean ± standard deviation unless otherwise stated. Comparisons between two groups were performed using a two-tailed unpaired Student’s *t*-test. Comparisons among three or more groups were performed using one-way analysis of variance followed by Tukey’s multiple comparison test. A *p*-value < 0.05 was considered statistically significant. No data points were excluded from statistical analysis unless they met the predefined exclusion criteria described above.

## 3. Results

### 3.1. Single-Cell Profiling Identifies Stromal Remodeling and Progenitor Differentiation Blockade in Nonunion

**Figure 1:**
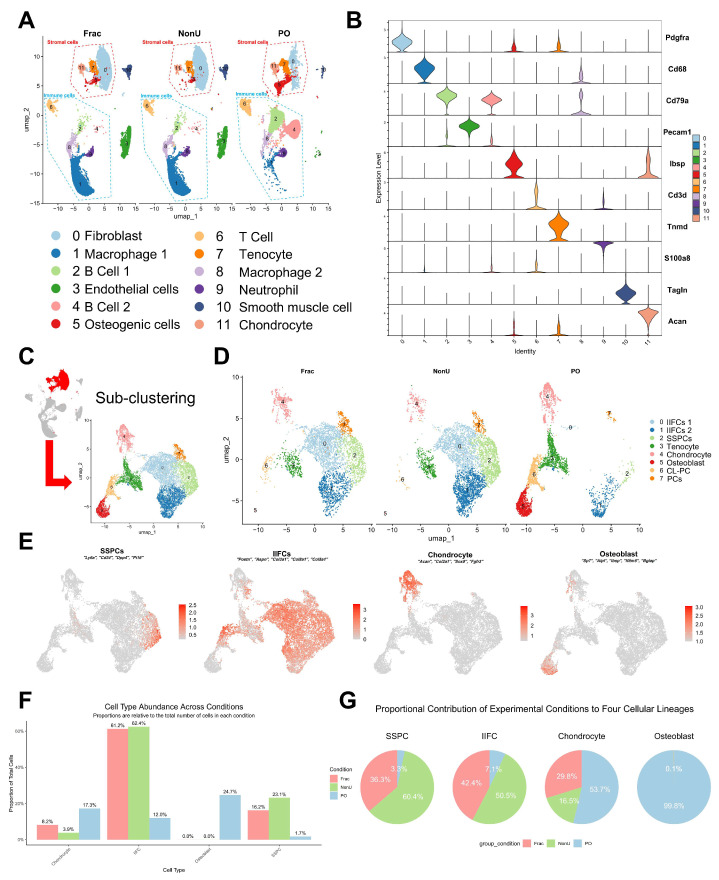
Single-cell profiling identifies stromal remodeling and progenitor differentiation blockade in fracture nonunion. (**A**) UMAP visualization of 12 major cell populations identified across periosteum (PO), normal fracture healing (Frac), and fracture nonunion (NonU) samples. (**B**) Violin plots showing the expression of canonical marker genes used to annotate the 12 major cell populations. (**C**) Schematic workflow showing isolation of the non-immune and non-vascular stromal compartment for sub-clustering analysis. (**D**) UMAP visualization of eight stromal subsets, colored by cell identity and split by experimental condition. (**E**) Feature plots showing module scores for SSPC, injury-induced fibrogenic cell (IIFC), chondrocyte, and osteoblast lineage programs in the stromal compartment. (**F**) Bar plot showing the relative abundance of SSPCs, IIFCs, chondrocytes, and osteoblasts among stromal cells in each experimental condition. (**G**) Pie charts showing the proportional contribution of PO, Frac, and NonU samples to each major stromal lineage population.

To characterize cellular alterations associated with fracture nonunion, we analyzed scRNA-seq data from fracture nonunion **(NonU)** and normal fracture healing **(Frac)** models, in which callus tissues were collected at 4 and 7 days post-fracture. Uninjured periosteal tissue was included as the periosteum **(PO)** baseline control. After quality control and doublet removal, 47,549 high-quality cells were retained for downstream analysis, including 12,788 cells from the NonU group, 14,022 cells from the Frac group, and 20,739 cells from the PO group (Figure S1A,B). After batch correction with Harmony, cells were integrated for unsupervised clustering and UMAP visualization (Figure 1A).

Using canonical marker genes, we annotated 12 major cell populations across three broad compartments: stromal cells, immune cells, and vascular cells (Figure 1A,B and Figure S1C) [45,46,47]. Because this study focused on stromal cell remodeling during nonunion, non-immune and non-vascular stromal cells were isolated for sub-clustering (Figure 1C). Based on canonical markers and a previously reported fracture cell atlas [21], the stromal compartment was further divided into eight subsets: IIFCs_1 and IIFCs_2, marked by *Postn*, *Aspn*, and *Col3a1*; SSPCs, marked by *Pi16*, *Dpp4*, and *Cd34*; tenocytes, marked by *Tnmd* and *Kera*; chondrocytes, marked by *Col2a1* and *Acan*; osteoblasts, marked by *Bglap* and *Ifitm5*; cambium-layer periosteal cells, marked by *Spp1* and *Postn*; and proliferating cells, marked by *Plk1* and *Mki67* (Figure 1D and Figure S1D). Lineage module scores further supported the annotation of SSPC, IIFC, chondrocyte, and osteoblast populations (Figure 1E).

Cell composition analysis indicated distinct stromal remodeling patterns across the three conditions. Compared with PO tissue, both Frac and NonU samples showed expansion of SSPCs and IIFCs, consistent with activation of periosteal stromal populations after injury (Figure 1F). Compared with the Frac group, the NonU group showed a higher proportion of SSPCs and IIFCs and a lower proportion of chondrocytes and osteoblasts within the stromal compartment (Figure 1F,G). This distribution was consistent with accumulation of progenitor and intermediate stromal states and reduced progression toward osteochondrogenic lineages in the nonunion microenvironment.

### 3.2. Nonunion-Associated Stromal Progenitors Display Coupled Ferroptosis-Associated and Pro-Inflammatory Signatures

**Figure 2:**
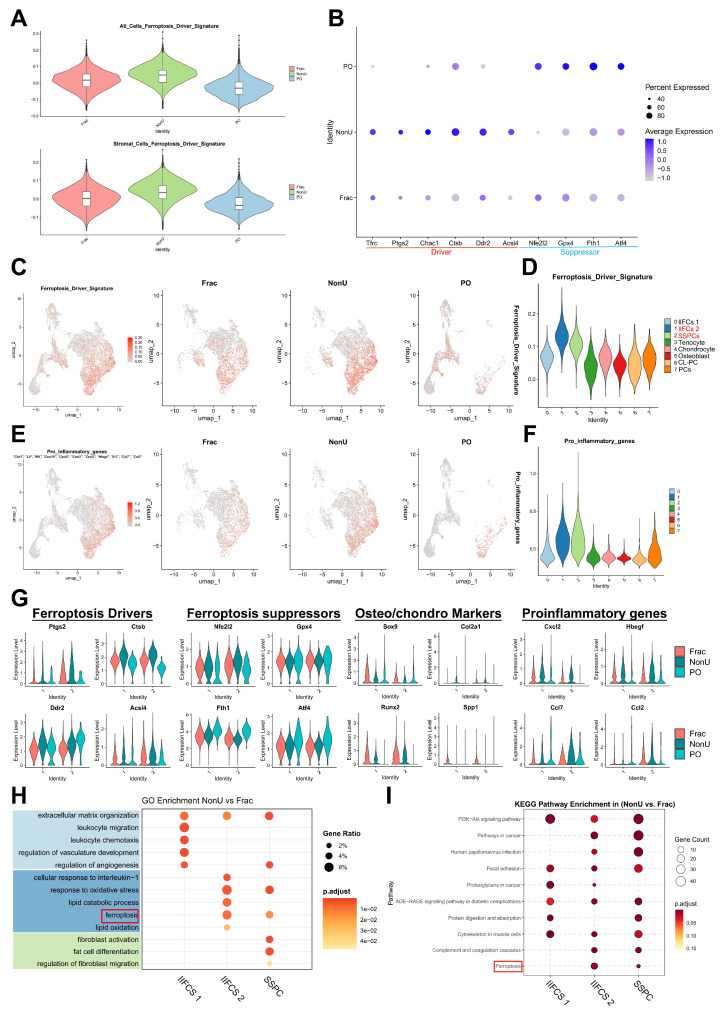
Nonunion-associated stromal progenitors display coupled pro-ferroptotic and pro-inflammatory signatures. (**A**) Violin plots comparing ferroptosis-driver module scores across experimental conditions in all cells and in the stromal compartment. (**B**) Dot plot showing the expression of representative ferroptosis driver and suppressor genes in stromal cells from PO, Frac, and NonU samples. (**C**) Feature plots showing the ferroptosis-driver module score in stromal cells, displayed overall and split by experimental condition. (**D**) Violin plots comparing ferroptosis-driver module scores across the eight stromal subsets. (**E**) Feature plots showing the pro-inflammatory module score in stromal cells, displayed overall and split by experimental condition. (**F**) Violin plots comparing pro-inflammatory module scores across the eight stromal subsets. (**G**) Violin plots showing representative ferroptosis driver genes, ferroptosis suppressor genes, osteochondrogenic markers, and pro-inflammatory genes in SSPCs and IIFCs_2 across experimental conditions. (**H**) GO enrichment analysis of genes upregulated in NonU versus Frac within SSPCs and IIFC subsets. Ferroptosis-related terms are highlighted. (**I**) KEGG enrichment analysis of genes upregulated in NonU versus Frac within SSPCs and IIFC subsets. The ferroptosis pathway is highlighted.

Ferroptosis-related gene signatures were then examined to identify molecular programs associated with the stromal differentiation block. The NonU group showed higher ferroptosis-driver module scores in both the total cell population and the stromal compartment (Figure 2A and Figure S2A,B). In stromal cells, representative ferroptosis driver genes, including *Acsl4*, *Ctsb*, and *Ptgs2*, were increased in NonU samples, whereas ferroptosis suppressor genes such as *Gpx4* and *Fth1* showed reduced expression (Figure 2B). In parallel, osteogenic and chondrogenic markers, including *Ibsp*, *Sp7*, *Acan*, and *Col2a1*, were reduced in the NonU group, whereas fibrosis-associated extracellular matrix genes, including *Col3a1* and *Col5a3*, were increased (Figure S2C).

Subset-level analysis showed that ferroptosis-driver module scores were highest in IIFCs_2 and SSPCs, particularly in the NonU condition (Figure 2C,D and Figure S2D). Ferroptosis suppressor genes did not show a consistent subset-specific downregulation pattern (Figure S2E,F). Because IIFCs have been reported to include inflammatory and reparative states [22], inflammatory gene programs were also assessed. The pro-inflammatory module score was enriched in IIFCs_2, overlapping with the subset that showed high ferroptosis-driver scores (Figure 2E,F). At the condition level, NonU samples showed higher pro-inflammatory and lower anti-inflammatory module scores than Frac samples (Figure S2G,H).

Marker-level analysis within SSPCs and IIFCs_2 further supported this coupled transcriptional state. In the NonU group, these subsets showed increased expression of ferroptosis-related and inflammatory genes together with reduced expression of osteogenic and chondrogenic markers (Figure 2G). GO and KEGG enrichment analyses of genes upregulated in NonU relative to Frac showed enrichment of inflammatory response, chemotaxis, extracellular matrix organization, and ferroptosis-related biological processes or pathways, particularly in SSPCs and IIFC subsets (Figure 2H,I). These data suggested that nonunion-associated stromal progenitors display a pro-inflammatory and pro-ferroptotic transcriptional state that coincides with reduced osteochondrogenic differentiation signatures.

### 3.3. Trajectory Analysis Suggests a Nonunion-Associated SSPC Fate Shift Toward a Ferroptosis-Associated State

**Figure 3:**
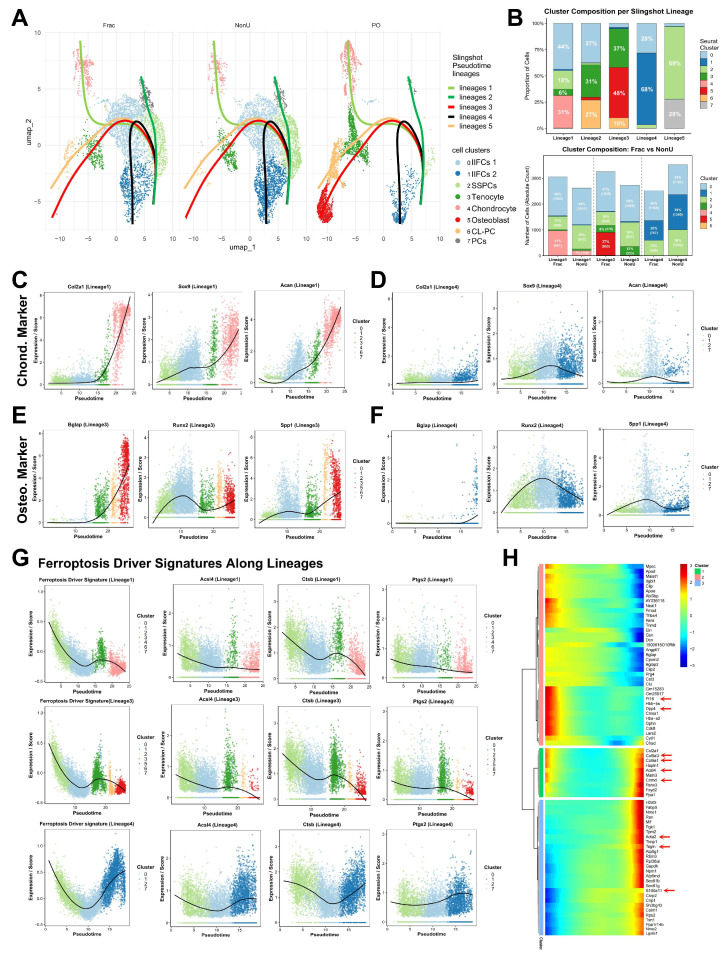
Pseudotime analysis suggests a nonunion-associated SSPC trajectory toward a ferroptosis-associated state. (**A**) Slingshot-inferred pseudotime lineages projected onto the stromal UMAP and displayed by experimental condition. (**B**) Stacked bar plots showing the cellular composition of the inferred lineages. The upper panel shows the relative proportion of stromal cell subsets within each lineage. The lower panel shows the distribution of Frac and NonU cells within Lineages 1, 3, and 4. (**C**) Scatter plots showing the expression of chondrogenic markers along the chondrogenic trajectory, Lineage 1, over pseudotime. (**D**) Scatter plots showing the expression of chondrogenic markers along the nonunion-associated trajectory, Lineage 4, over pseudotime. (**E**) Scatter plots showing the expression of osteogenic markers along the osteogenic trajectory, Lineage 3, over pseudotime. (**F**) Scatter plots showing the expression of osteogenic markers along the nonunion-associated trajectory, Lineage 4, over pseudotime. (**G**) Scatter plots showing the ferroptosis-driver module score and representative ferroptosis driver genes, including *Acsl4*, *Ctsb*, and *Ptgs2*, along Lineage 3, Lineage 1, and Lineage 4. (**H**) Heatmap showing dynamic gene expression changes across cells ordered along Lineage 4. Colors indicate row-scaled gene expression levels, as shown by the color scale. Red arrows indicate representative genes highlighted in the analysis, including SSPC-associated markers, ferroptosis-related genes, and pro-fibrotic markers.

To examine stromal fate transitions during fracture repair, we performed Slingshot pseudotime analysis and inferred five putative lineages with SSPCs assigned as the starting population (Figure 3A). Lineage 1 and Lineage 3 were consistent with chondrogenic and osteogenic differentiation trajectories, respectively. Lineage 4 represented a distinct trajectory enriched for IIFCs_2, which accounted for 68% of the cells in this lineage (Figure 3B).

Lineage composition analysis showed different distributions between the Frac and NonU groups. Frac cells were preferentially represented in the chondrogenic and osteogenic trajectories, whereas NonU cells were less abundant in these regenerative lineages and accumulated more prominently in Lineage 4 (Figure 3B). This pattern was consistent with reduced osteochondrogenic progression and increased representation of an alternative stromal state under nonunion conditions.

We next examined marker dynamics along pseudotime. Chondrogenic markers, including *Acan*, *Sox9*, and *Col2a1*, progressively increased along Lineage 1 (Figure 3C), while osteogenic markers, including *Bglap*, *Runx2*, and *Spp1*, increased along Lineage 3 (Figure 3E). In contrast, these regenerative markers were reduced along Lineage 4 (Figure 3D,F). Representative ferroptosis driver genes, including *Acsl4*, *Ctsb*, and *Ptgs2*, increased along Lineage 4 but remained low or decreased along the chondrogenic and osteogenic trajectories (Figure 3G). In parallel, the ferroptosis suppressor signature and additional ferroptosis-related regulators, including Gpx4, Ncoa4, and Aifm2, showed distinct dynamic patterns across Lineages 1, 3, and 4, with modest changes along the pseudotime trajectories (Figure S3). Heatmap analysis further showed that cells ordered along Lineage 4 gradually lost SSPC-associated markers, including *Pi16* and *Dpp4*, and acquired increased ferroptosis-related and pro-fibrotic gene expression (Figure 3H). These data suggested that a subset of nonunion-associated SSPCs was positioned along an alternative IIFC-enriched trajectory associated with a ferroptosis-related transcriptional state.

### 3.4. SCENIC Analysis Identifies Candidate Transcription Factors Associated with the Ferroptosis-Associated Trajectory

**Figure 4:**
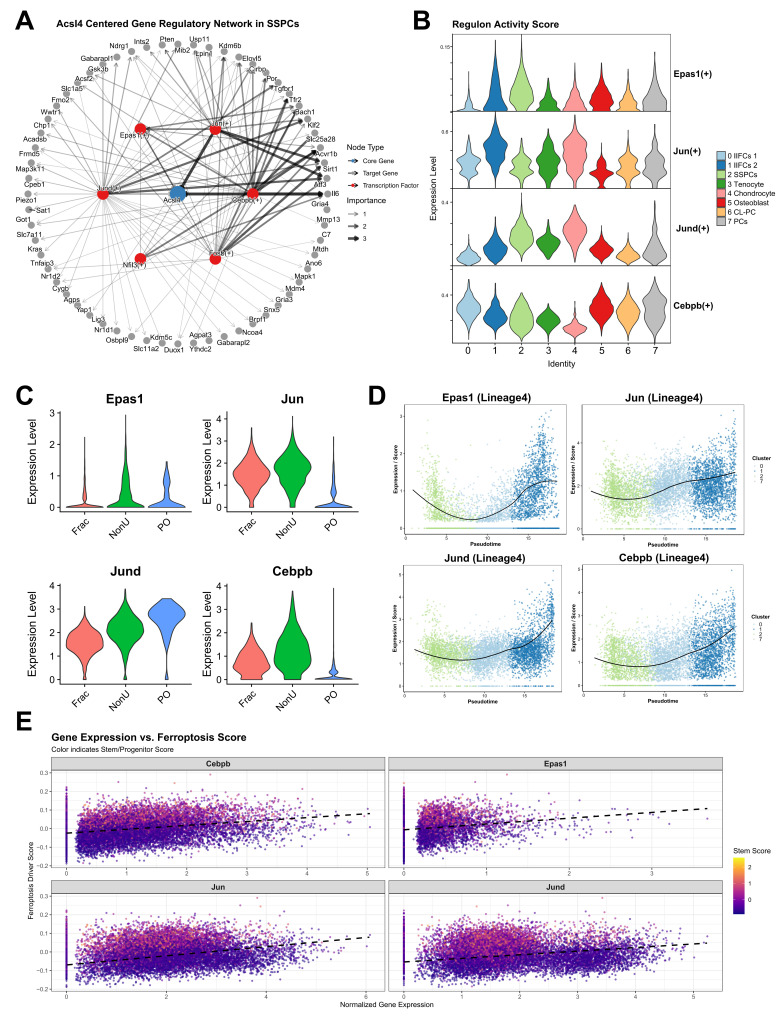
Gene regulatory network analysis identifies candidate transcription factors associated with the ferroptosis-associated stromal state. (**A**) SCENIC-inferred transcription factor regulatory network centered on *Acsl4* in SSPCs. (**B**) Violin plots showing SCENIC regulon activity scores for the candidate transcription factors *Jun*, *Jund*, *Cebpb*, and *Epas1* across the identified stromal cell subsets. (**C**) Violin plots showing the expression of *Jun*, *Jund*, *Cebpb*, and *Epas1* across experimental conditions. (**D**) Scatter plots showing the regulon activity of the candidate transcription factors along Lineage 4 pseudotime. (**E**) Scatter plots showing the correlation between candidate transcription factor regulon activity and ferroptosis score at the single-cell level. Each dot represents one cell and is colored by stemness score. Dashed lines indicate linear regression fits.

To identify transcriptional regulators associated with the ferroptosis-related stromal trajectory, we performed SCENIC analysis in SSPCs. Because *Acsl4* was enriched in the nonunion-associated ferroptosis-related state, we examined its predicted upstream regulatory network and visualized the corresponding transcription factor sub-network (Figure 4A; Supplementary File S1). Based on regulon structure and activity, *Jun*, *Jund*, *Cebpb*, and *Epas1* were selected as candidate regulators for further analysis.

SCENIC regulon activity analysis showed that these four transcription factors were preferentially active in SSPCs and related stromal subsets (Figure 4B). Direct gene expression analysis further showed higher expression of *Jun*, *Jund*, *Cebpb*, and *Epas1* in the NonU group than in the Frac group (Figure 4C). Along Lineage 4 pseudotime, the regulon activity of these transcription factors increased progressively (Figure 4D).

We next examined the relationship between candidate transcription factor activity and ferroptosis-related features at the single-cell level. Regulon activity of *Jun*, *Jund*, *Cebpb*, and *Epas1* showed positive associations with the ferroptosis score, with positive slopes in the fitted regression lines (Figure 4E). Cells with relatively higher stemness scores were also distributed within the high-ferroptosis-score region (Figure 4E). These findings nominated *Jun*, *Jund*, *Cebpb*, and *Epas1* as candidate transcriptional regulators associated with the nonunion-related, ferroptosis-associated stromal state.

### 3.5. Intercellular Communication Analysis Prioritizes IL-1β as a Candidate Upstream Signal Potentially Associated with Neutrophils

**Figure 5:**
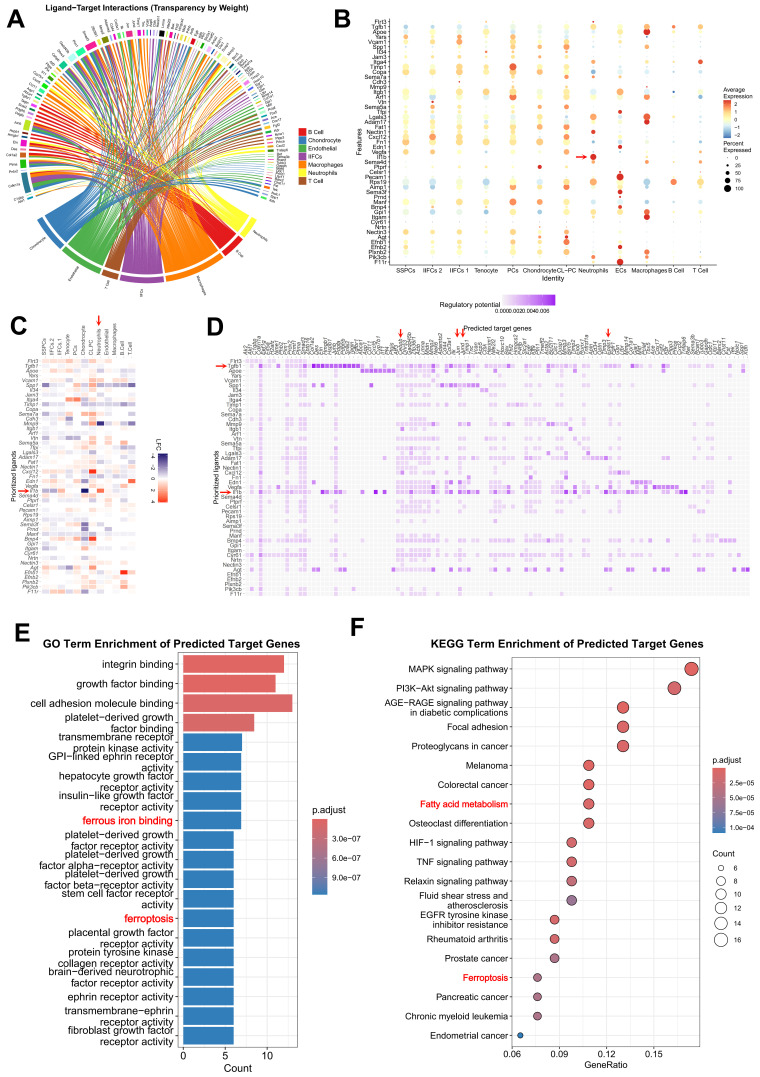
Intercellular communication analysis predicts IL-1β as a candidate upstream signal potentially associated with neutrophils. (**A**) Chord diagram showing predicted ligand–target interactions between sender cell populations and receiver SSPCs in the NonU microenvironment. Immune cell populations contributed prominently to the inferred SSPC-directed signaling network. (**B**) Dot plot showing the top predicted ligands received by SSPCs and their expression patterns across potential sender cell populations. Dot size indicates the percentage of ligand-expressing cells, and color indicates average expression. (**C**) Heatmap showing log-fold changes in prioritized ligands in their corresponding sender populations when comparing NonU with Frac conditions. (**D**) Heatmap showing the predicted regulatory potential of prioritized ligands on SSPC target genes. Candidate transcription factors are indicated. (**E**) GO enrichment analysis of predicted IL-1β target genes in SSPCs. Ferroptosis-related terms are highlighted. (**F**) KEGG enrichment analysis of predicted IL-1β target genes in SSPCs. Ferroptosis and fatty acid metabolism pathways are highlighted. Red arrows and red-colored labels indicate representative ligands, cell populations, target genes, transcription factors, or enriched terms highlighted and discussed in the main text.

Having identified candidate transcription factors associated with the ferroptosis-related SSPC trajectory, we next investigated extracellular signals that might regulate this pathological stromal state. NicheNet analysis was performed using SSPCs as the receiver population and all annotated cell populations as potential senders. A global ligand–target interaction network showed extensive predicted communication between sender populations and SSPCs in the NonU microenvironment. Notably, immune cell populations contributed prominently to the inferred SSPC-directed signaling network (Figure 5A).

We next visualized the top predicted ligands received by SSPCs together with their expression patterns across potential sender cell populations. Among these ligands, *Il1b* showed an immune-cell-associated expression pattern and was prominently enriched in neutrophils, suggesting neutrophils as a potential source of IL-1β-mediated inflammatory input to SSPCs (Figure 5B). Comparison between NonU and Frac conditions further showed that *Il1b* was one of the most strongly upregulated prioritized ligands in its source population, particularly neutrophils (Figure 5C). Thus, IL-1β was prioritized because it was predicted to act on SSPCs, showed immune-cell enrichment, and was upregulated in the NonU microenvironment.

We next examined the predicted ligand–target regulatory relationships in SSPCs. NicheNet linked several candidate ligands, including *Il1b*, *Apoe*, *Vegfa*, and *Tgfb1*, to transcriptional responses in SSPCs (Figure 5D). Among these, *Il1b* showed predicted regulatory potential toward target genes related to the candidate transcriptional program identified above, including *Jun*, *Jund*, *Cebpb*, and *Epas1*. Given its neutrophil-enriched expression and NonU-associated upregulation, IL-1β was selected as the leading immune-derived candidate for downstream analysis.

Functional enrichment analysis of predicted IL-1β target genes in SSPCs showed enrichment of terms related to growth factor activity, ligand binding, ferrous iron binding, and ferroptosis-related biological processes (Figure 5E). KEGG analysis of these predicted IL-1β target genes further showed enrichment of ferroptosis, fatty acid metabolism, and inflammation- or stress-related pathways (Figure 5F). These results suggested that IL-1β, potentially derived in part from neutrophils, may be linked to iron handling, lipid metabolism, and ferroptosis-related transcriptional changes in SSPCs.

PROGENy analysis further showed increased NF-κB, MAPK, and TNF-α pathway activities in NonU stromal cells, including SSPCs and IIFC populations (Figure S4A,B). NF-κB activity was positively associated with candidate transcription factor expression, including *Epas1* and *Jun*, particularly in cells with higher ferroptosis-driver scores (Figure S4C). Together, these findings prioritized IL-1β as a candidate upstream immune signal, with neutrophils representing a potential contributing source, associated with the NF-κB/EPAS1-related ferroptosis program in SSPCs.

### 3.6. IL-1β Induces Ferroptotic Stress and Impairs SSPC Function In Vitro

**Figure 6:**
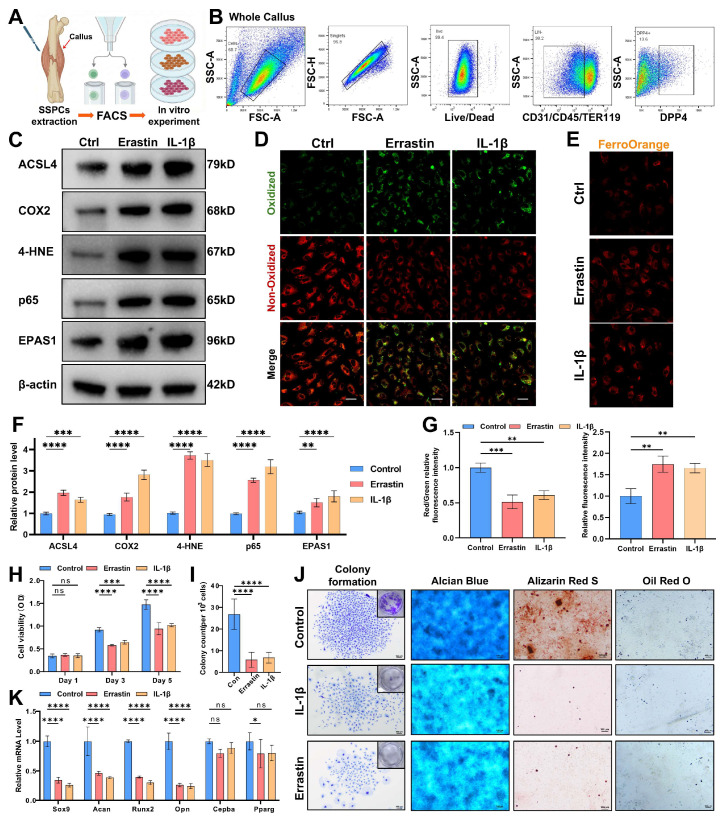
IL-1β induces ferroptotic stress and impairs SSPC function in vitro. (**A**) Schematic overview of SSPC isolation by fluorescence-activated cell sorting and subsequent in vitro validation experiments. (**B**) Flow cytometry gating strategy for isolating Lin^−^/DPP4^+^ SSPCs from mouse callus tissue at 7 days post-fracture. (**C**) Representative Western blot images showing the expression of ACSL4, COX2, 4-HNE, NF-κB p65, and EPAS1 in SSPCs treated with vehicle, erastin, or IL-1β. β-actin was used as the loading control. (**D**) Quantification of Western blot protein expression levels shown in panel C (*n* = 3). (**E**) Representative BODIPY 581/591 C11 fluorescence images showing lipid peroxidation in SSPCs. Oxidized lipids are indicated by increased green fluorescence and reduced red fluorescence. Scale bar = 50 μm. (**F**) Representative FerroOrange fluorescence images showing intracellular Fe^2+^ levels in SSPCs. Scale bar = 50 μm. (**G**) Quantification of the red/green fluorescence intensity ratio from BODIPY 581/591 C11 staining and the relative FerroOrange fluorescence intensity (*n* = 3). A lower red/green ratio indicates increased lipid peroxidation. (**H**) Quantification of colony-forming unit efficiency and cell viability measured by CCK-8 assay (*n* = 3). (**I**) qRT-PCR analysis of osteogenic, chondrogenic, and adipogenic marker gene expression (*n* = 3). (**J**) Representative images of tri-lineage differentiation assays, including Alizarin Red S staining for osteogenesis, Alcian Blue staining for chondrogenesis, and Oil Red O staining for adipogenesis. Scale bar = 200 μm. (**K**) qRT-PCR analysis of osteogenic, chondrogenic, and adipogenic marker gene expression, including Runx2, Opn, Acan, Col2a1, Pparg, and Cebpa (*n* = 3). Data are presented as mean ± SD. Statistical significance was determined by one-way ANOVA followed by Tukey’s multiple comparison test. ns, not significant; * *p* < 0.05, ** *p* < 0.01, *** *p* < 0.001, **** *p* < 0.0001.

To examine the effect of IL-1β on SSPCs, primary SSPCs were isolated from mouse callus tissue by fluorescence-activated cell sorting using a Lin^−^/DPP4^+^ gating strategy (Figure 6A,B). Sorted SSPCs were treated with vehicle, erastin, or IL-1β, with erastin used as a positive control for ferroptosis induction. Western blot analysis showed that IL-1β increased the levels of ACSL4 and COX2, together with the lipid peroxidation marker 4-HNE, the inflammatory signaling component NF-κB p65, and the transcription factor EPAS1 (Figure 6C,D). This pattern was consistent with activation of a ferroptosis-associated response and the predicted NF-κB/EPAS1-related program.

We next assessed lipid peroxidation and intracellular Fe^2+^ accumulation. BODIPY 581/591 C11 staining showed a reduced red/green fluorescence intensity ratio in IL-1β-treated SSPCs, consistent with increased lipid peroxidation (Figure 6E,G). FerroOrange staining showed increased intracellular Fe^2+^ levels after IL-1β treatment (Figure 6F,G). These changes were similar in direction to those observed after erastin treatment. Fer-1 co-treatment further attenuated IL-1β-induced lipid peroxidation, intracellular Fe^2+^ accumulation, and ferroptosis-related protein changes, supporting the involvement of ferroptotic stress in the IL-1β-induced SSPC response (Figure S5A–F).

We then evaluated SSPC self-renewal and differentiation capacity. Both IL-1β and erastin reduced colony-forming efficiency and cell viability compared with vehicle control (Figure 6H,I). Consistently, tri-lineage differentiation assays showed reduced Alizarin Red S, Alcian Blue, and Oil Red O staining after IL-1β exposure (Figure 6J). qRT-PCR analysis showed that IL-1β decreased the expression of osteogenic markers *Runx2* and *Opn*, chondrogenic markers *Acan* and *Col2a1*, and adipogenic markers *Pparg* and *Cebpa* (Figure 6K). These results suggested that IL-1β induced ferroptotic stress in primary SSPCs and was associated with impaired self-renewal and differentiation capacity in vitro.

### 3.7. EPAS1 Inhibition Attenuates IL-1β-Induced Ferroptotic Stress and Differentiation Impairment in SSPCs

**Figure 7:**
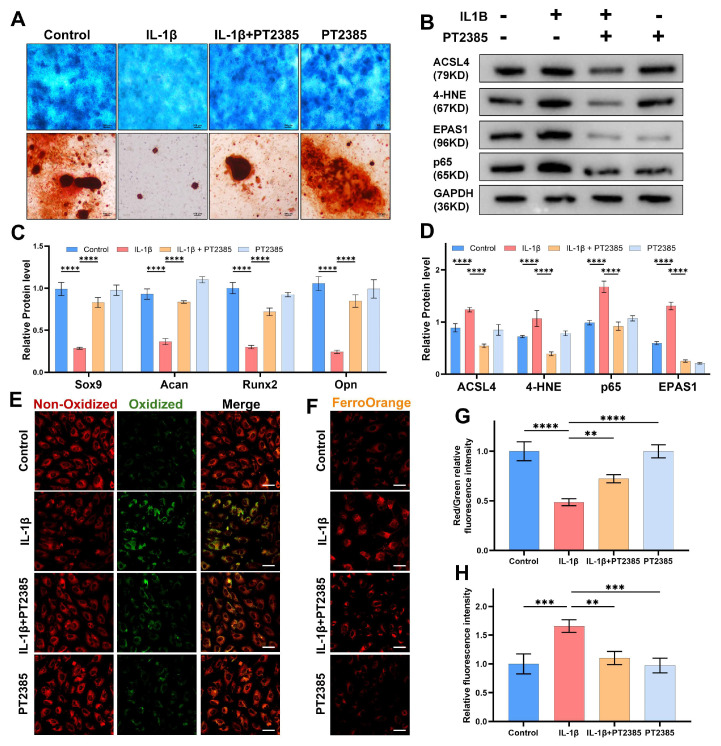
Pharmacological EPAS1 inhibition attenuates IL-1β-induced ferroptotic stress and differentiation impairment in primary SSPCs. (**A**) Representative images of Alcian Blue and Alizarin Red S staining in primary SSPCs treated with vehicle control, IL-1β, IL-1β plus PT2385, or PT2385 alone. Scale bars = 200 μm. (**B**) Representative Western blot images showing ACSL4, 4-HNE, EPAS1, and NF-κB p65 protein levels in SSPCs under the indicated treatments. GAPDH was used as the loading control. (**C**) qRT-PCR analysis of differentiation-related marker genes, including the chondrogenic markers *Sox9* and *Acan* and the osteogenic markers *Runx2* and *Opn* (n = 3). (**D**) Quantification of Western blot protein expression levels shown in panel (**B**) (n = 3). (**E**) Representative BODIPY 581/591 C11 fluorescence images showing lipid peroxidation in SSPCs. Red fluorescence indicates non-oxidized lipid signal, and green fluorescence indicates oxidized lipid signal. Scale bars = 50 μm. (**F**) Representative FerroOrange fluorescence images showing intracellular Fe^2+^ levels in SSPCs under the indicated treatments. Scale bars = 50 μm. (**G**) Quantification of the red/green fluorescence intensity ratio from BODIPY 581/591 C11 staining (n = 3). A lower red/green ratio indicates increased lipid peroxidation. (**H**) Quantification of relative FerroOrange fluorescence intensity (n = 3). Data are presented as mean ± SD. Statistical significance was determined by one-way ANOVA followed by Tukey’s multiple comparison test. ** *p* < 0.01, *** *p* < 0.001, **** *p* < 0.0001.

To test whether EPAS1 inhibition could attenuate IL-1β-induced SSPC dysfunction, primary SSPCs were treated with vehicle, IL-1β, IL-1β plus PT2385, or PT2385 alone. In differentiation assays, IL-1β reduced Alcian Blue and Alizarin Red S staining, indicating impaired chondrogenic and osteogenic differentiation. PT2385 co-treatment partially restored both staining signals, whereas PT2385 alone did not show an obvious inhibitory effect under basal conditions (Figure 7A).

Consistent with the staining results, qRT-PCR analysis showed that IL-1β significantly decreased the expression of chondrogenic markers *Sox9* and *Acan* and osteogenic markers *Runx2* and *Opn*. These reductions were attenuated by PT2385 co-treatment (Figure 7C). Western blot analysis further showed that IL-1β increased ACSL4, 4-HNE, EPAS1, and NF-κB p65 protein levels. Compared with IL-1β treatment alone, PT2385 co-treatment reduced EPAS1 expression and lowered the levels of ACSL4, 4-HNE, and p65 (Figure 7B,D).

We next examined lipid peroxidation and intracellular Fe^2+^ accumulation. BODIPY 581/591 C11 staining showed that IL-1β decreased the red/green fluorescence intensity ratio, consistent with increased lipid peroxidation. PT2385 co-treatment partially restored the red/green ratio (Figure 7E,G). FerroOrange staining showed that IL-1β increased intracellular Fe^2+^ levels, whereas PT2385 co-treatment reduced this increase (Figure 7F,H). These findings support that pharmacological EPAS1 inhibition with PT2385 attenuates IL-1β-induced ferroptotic stress and partially restores SSPC osteochondrogenic differentiation.

### 3.8. Pharmacological EPAS1 Inhibition Attenuates IL-1β-Induced Impairment of Bone Regeneration In Vivo

**Figure 8:**
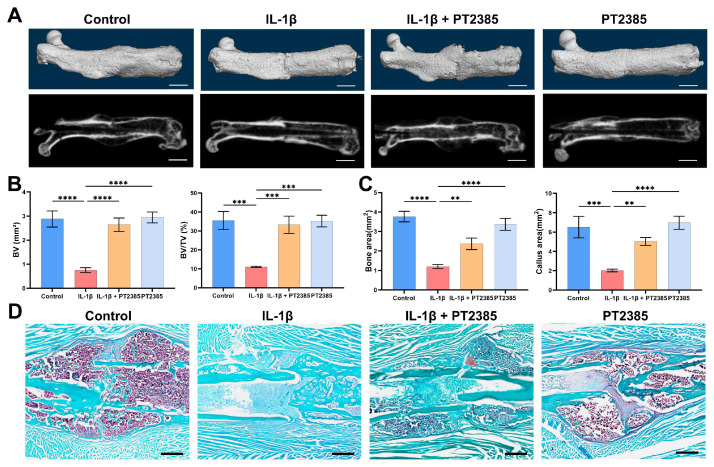
EPAS1 inhibition attenuates IL-1β-impaired bone regeneration in vivo. (**A**) Representative micro-CT three-dimensional reconstructions and micro-CT sectional images of fractured mouse femurs from vehicle control, IL-1β, IL-1β plus PT2385, and PT2385 alone groups at 28 days post-fracture. Scale bars = 1 mm. (**B**) Quantitative micro-CT analysis of bone volume (BV) and bone volume fraction (BV/TV) in the fracture callus region. (**C**) Quantitative histomorphometric analysis of bone area and callus area among the four treatment groups. (**D**) Representative Safranin O/Fast Green staining images of the fracture callus region showing callus organization, cartilage matrix, and newly formed bone tissue under the indicated treatments. Scale bars = 500 μm. Data are presented as mean ± SD; *n* = 6 mice per group. Statistical significance was determined by one-way ANOVA followed by Tukey’s multiple comparison test. ** *p* < 0.01, *** *p* < 0.001, **** *p* < 0.0001.

To assess whether EPAS1 inhibition could mitigate IL-1β-impaired fracture repair in vivo, mice were assigned to vehicle control, IL-1β, IL-1β plus PT2385, or PT2385 alone groups and analyzed at 28 days post-fracture. Micro-CT reconstruction showed continuous mineralized callus formation in the control group. IL-1β-treated mice showed reduced mineralized callus formation and poorer structural continuity at the fracture site, whereas PT2385 co-treatment improved callus architecture and mineralized tissue formation (Figure 8A). PT2385 alone did not show an obvious adverse effect on fracture repair.

Quantitative micro-CT analysis showed that IL-1β significantly reduced bone volume and BV/TV within the fracture callus region. Both parameters were increased in the IL-1β plus PT2385 group compared with the IL-1β group (Figure 8B). Histomorphometric analysis showed a similar pattern: IL-1β reduced bone area and callus area, while PT2385 co-treatment partially restored both indices (Figure 8C). Safranin O/Fast Green staining further showed poor callus organization and reduced newly formed bone matrix in the IL-1β group. In contrast, PT2385 co-treatment was associated with improved callus structure and matrix formation (Figure 8D). These results suggest that pharmacological EPAS1 inhibition attenuates IL-1β-associated impairment of bone regeneration in vivo.

### 3.9. Mendelian Randomization Provides Exploratory Genetic Support Linking IL-1β and EPAS1 to Human Bone Nonunion Risk

To explore the human genetic relevance of the IL-1β/EPAS1-associated program, we performed two-sample Mendelian randomization using exposure summary statistics from OpenGWAS and bone nonunion outcome data from FinnGen. The exposure datasets included *EPAS1* expression (N = 31,470) and circulating IL-1β protein levels (N = 997), while the bone nonunion outcome dataset included 212,626 individuals. After instrument selection and linkage disequilibrium clumping, 35 independent SNPs were retained for *EPAS1* and 7 independent SNPs for IL-1β. The mean F-statistics were 42.6 for *EPAS1* and 17.7 for IL-1β, indicating adequate instrument strength (Figure S6A,B; Supplementary File S2).

**Figure 9:**
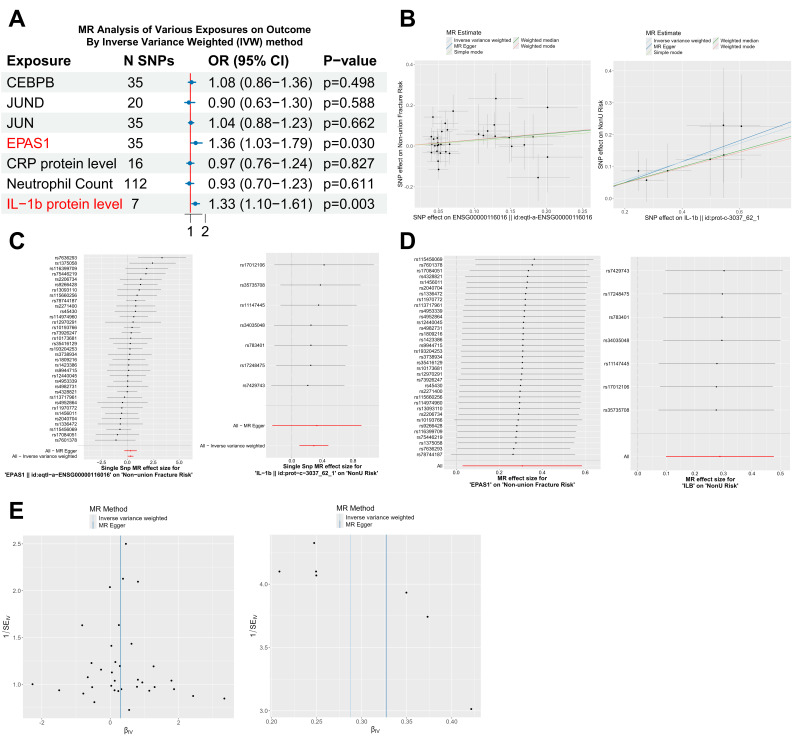
Mendelian randomization provides exploratory genetic support linking IL-1β and *EPAS1* to human bone nonunion risk. (**A**) Forest plot summarizing inverse-variance weighted Mendelian randomization estimates for the tested exposures and bone nonunion risk. Odds ratios and 95% confidence intervals are shown. (**B**) Scatter plots showing the associations between SNP effects on *EPAS1* expression or IL-1β levels and SNP effects on bone nonunion risk. Lines indicate estimates from different Mendelian randomization methods. (**C**) Single-SNP forest plots showing individual SNP estimates for *EPAS1* and IL-1β. The combined inverse-variance weighted estimates are shown at the bottom. (**D**) Leave-one-out analyses for *EPAS1* and IL-1β, performed by sequentially excluding one SNP at a time. (**E**) Funnel plots used to assess potential directional pleiotropy in the Mendelian randomization analyses for *EPAS1* and IL-1β. Red colors, red lines, and numbers indicate representative annotations used to highlight key features discussed in the main text.

In the primary inverse-variance weighted analysis, genetically predicted higher *EPAS1* expression was associated with increased bone nonunion risk (OR = 1.35, 95% CI: 1.02–1.78, *p* = 0.038). Higher genetically predicted circulating IL-1β levels were also associated with increased bone nonunion risk (OR = 1.33, 95% CI: 1.10–1.61, *p* = 0.003) (Figure 9A). Complementary MR methods, including weighted median and MR-Egger analyses, showed effect estimates in the same direction as the inverse-variance weighted analysis (Supplementary File S2). In contrast, no significant associations were observed for *CEBPB*, *JUN*, *JUND*, C-reactive protein, or blood neutrophil count (Figure 9A; Supplementary File S2).

Sensitivity analyses supported the stability of the associations for *EPAS1* and IL-1β. Scatter plots and single-SNP forest plots showed generally consistent directional effects across instruments (Figure 9B,C). Leave-one-out analysis indicated that the estimates were not driven by a single SNP (Figure 9D). Funnel plots did not show obvious asymmetry (Figure 9E). Quantitative sensitivity analyses showed no evidence of substantial heterogeneity for *EPAS1* (Cochran’s Q *p* = 0.28) or IL-1β (Cochran’s Q *p* = 0.99), and MR-Egger intercept tests did not indicate horizontal pleiotropy (*EPAS1*, intercept *p* = 0.99; IL-1β, intercept *p* = 0.89) (Supplementary File S2).

Reverse MR analysis was then performed to assess whether genetic liability to bone nonunion was associated with IL-1β or *EPAS1*. Using 28 SNPs associated with bone nonunion, no significant reverse association was observed for IL-1β levels (IVW: OR = 1.04, *p* = 0.437). Sensitivity analyses showed no evidence of substantial heterogeneity or directional pleiotropy (Figure S6C–F; Supplementary File S3). For *EPAS1*, only one valid instrument was available, and Wald ratio analysis did not support a significant reverse association (β = −0.001, *p* > 0.05) (Supplementary File S3). Together, these findings provided exploratory genetic evidence consistent with potential links between IL-1β, EPAS1, and human bone nonunion risk. Given the relatively limited sample size of the circulating IL-1β GWAS, the IL-1β-related MR result should be interpreted with caution.

## 4. Discussion

This study identified an IL-1β/EPAS1-associated ferroptotic program linked to SSPC dysfunction in inflammation-associated fracture nonunion. Across single-cell analyses, functional experiments, and exploratory human genetic evidence, nonunion-associated SSPCs and IIFC subsets showed a coupled pro-inflammatory and pro-ferroptotic state. IL-1β treatment induced lipid peroxidation, intracellular Fe^2+^ accumulation, ferroptosis-related protein expression, and impaired SSPC differentiation in vitro. Fer-1 further attenuated IL-1β-induced lipid peroxidation, Fe^2+^ accumulation, and ferroptosis-related molecular changes, providing additional inhibitor-based support for the involvement of ferroptotic stress. Pharmacological EPAS1 inhibition attenuated IL-1β-induced ferroptotic stress and improved IL-1β-impaired fracture repair in vivo. These findings support a model in which inflammatory signaling contributes to diversion of SSPCs from osteochondrogenic repair toward an EPAS1-related ferroptotic state.

The single-cell analysis extends current knowledge of stromal remodeling during fracture repair. Previous work has shown that fracture healing depends on periosteal SSPCs and their transition through an injury-induced fibrogenic cell state before progression toward osteogenic and chondrogenic lineages [19,20,21,22]. Recent single-cell studies further indicate that stromal cell states are dynamically shaped by the immune microenvironment during repair and musculoskeletal trauma [21,22]. In the present study, the NonU condition was not characterized by a simple loss of stromal progenitors. Instead, SSPCs and IIFCs accumulated, whereas chondrocytes and osteoblasts were reduced. Pseudotime analysis further suggested that a subset of SSPCs preferentially entered an IIFC-enriched trajectory with increased ferroptosis-related and pro-fibrotic gene expression. This pattern is consistent with previous observations that prolonged inflammatory stromal and immune states contribute to fibrotic repair failure [29,30], and adds ferroptosis-associated stress as a potential cell-intrinsic feature of the abnormal IIFC-like state.

Ferroptosis provides a plausible link between inflammatory stress and progenitor dysfunction. Ferroptosis is an iron-dependent regulated cell death process driven by lipid peroxidation, and its interaction with inflammation has been increasingly recognized in degenerative and immune-mediated diseases [23,24,48,49]. In orthopedic disease contexts, ferroptosis has been implicated in osteoarthritis, intervertebral disc degeneration, and other bone-related disorders [25,26,27,28]. Several reports also indicate that inflammatory stimuli, including IL-1β, can promote oxidative stress, lipid ROS accumulation, and ferroptosis-related changes in chondrocytes or skeletal lineage-related cells [50,51,52]. These studies support the biological plausibility of our finding that IL-1β induced lipid peroxidation, Fe^2+^ accumulation, ACSL4/COX2 upregulation, and 4-HNE accumulation in primary SSPCs. Importantly, our data place this ferroptosis-associated response within the fracture nonunion niche and link it to impaired SSPC self-renewal and osteochondrogenic differentiation.

The computational analyses provided a candidate regulatory framework connecting inflammatory ligands to ferroptosis-associated SSPC dysfunction. NicheNet prioritized IL-1β as a candidate upstream ligand targeting SSPCs, with neutrophils representing a potential contributing source, while PROGENy analysis showed increased NF-κB-related pathway activity in NonU stromal cells. This is consistent with the established role of NF-κB signaling as a core inflammatory pathway and with prior work showing that IL-1β and TNF-α can alter stromal, vascular, and skeletal lineage cell behavior under inflammatory conditions [15,16,17,18]. SCENIC analysis further identified Jun, Jund, Cebpb, and Epas1 as candidate transcriptional regulators associated with the ferroptosis-enriched trajectory. Among these factors, Epas1 was prioritized because its regulon activity increased along the nonunion-associated trajectory and because pharmacological EPAS1 inhibition attenuated IL-1β-induced ferroptotic stress. HIF-2α, encoded by Epas1, has been reported as a stress-responsive and catabolic transcriptional regulator in cartilage pathology, where it contributes to matrix degradation and osteoarthritis progression [53,54]. Therefore, the IL-1β/NF-κB/EPAS1 link observed in our study is consistent with prior evidence connecting inflammatory signaling, HIF-2α/EPAS1 activity, and skeletal tissue degeneration. However, NicheNet and SCENIC results remain regulatory predictions and should not be interpreted as direct evidence of ligand–target or transcription factor–target binding. Because this inference was based on transcriptomic ligand–target modeling, future in vivo validation will be needed to determine the relative contribution of neutrophils and other immune or stromal populations.

The functional rescue experiments strengthened the proposed relationship between IL-1β, EPAS1, and ferroptotic SSPC dysfunction. PT2385 is a pharmacological HIF-2α/EPAS1 inhibitor that disrupts HIF-2α activity and has been evaluated in HIF-2α-driven disease settings [55,56]. In primary SSPCs, pharmacological EPAS1 inhibition with PT2385 reduced IL-1β-associated EPAS1 expression and attenuated the increase in ACSL4, 4-HNE, p65, lipid peroxidation, and intracellular Fe^2+^. It also partially restored osteogenic and chondrogenic differentiation. In vivo, PT2385 improved mineralized callus formation and histological repair in IL-1β-treated fractured femurs. However, because PT2385 is a pharmacological inhibitor, these data support the functional involvement of EPAS1 but require future confirmation using EPAS1 knockdown, silencing, or SSPC-specific Epas1 loss-of-function models. These findings do not establish PT2385 as a clinical treatment for fracture nonunion, but they support EPAS1-associated ferroptotic stress as a candidate intervention point. Given that fracture repair is influenced by mechanical stability, vascularization, immune cell dynamics, and systemic comorbidities [3,6,7], future therapeutic exploration should evaluate EPAS1 inhibition within broader models that better approximate clinical nonunion biology.

From a translational perspective, current management of atrophic nonunion mainly relies on improving mechanical stability, debridement of fibrotic tissue, bone grafting, and biological stimulation [57,58]. Our findings do not replace these principles, but suggest that local inflammatory and ferroptosis-associated stress may represent an additional biological barrier to regeneration. Targeting EPAS1-related signaling or ferroptotic stress may therefore be considered as a potential adjunctive strategy [47,56]. However, several challenges remain, including treatment timing, local delivery, dose control, long-term safety, and possible effects of EPAS1 or ferroptosis modulation on vascular, immune, and skeletal cells within the repair niche [47,56].

The Mendelian randomization analysis provided exploratory human genetic support for the relevance of IL-1β and EPAS1 to bone nonunion risk. Genetically predicted higher circulating IL-1β levels and higher EPAS1 expression were associated with increased nonunion risk, whereas reverse MR did not support a clear reverse association. These results are aligned with the experimental model, but they should not be treated as definitive clinical causality. MR estimates depend on instrument strength, exposure definition, population structure, pleiotropy, and the clinical accuracy of the outcome phenotype. In particular, the GWAS dataset for circulating IL-1β had a relatively limited sample size (N = 997), which may reduce statistical power, increase uncertainty in instrument selection, and limit the generalizability of the IL-1β-related MR estimate. This is particularly relevant here because the available IL-1β protein dataset had a limited sample size and bone nonunion is a heterogeneous phenotype that may reflect different anatomical sites, mechanical environments, and comorbidities. Thus, the MR findings are best interpreted as population-level support for further investigation of the IL-1β/EPAS1-associated program in human fracture nonunion.

Several limitations should be considered. First, PT2385 is a pharmacological EPAS1 inhibitor and was not delivered specifically to SSPCs; effects on other cell types within the fracture microenvironment cannot be excluded. Progenitor-specific Epas1 loss-of-function models would be needed to define the cell-autonomous contribution of EPAS1 more precisely. In addition, the regulatory links inferred by SCENIC were not validated by chromatin-binding assays. Second, although ferroptosis-related changes were supported by transcriptomic signatures, lipid peroxidation, Fe^2+^ accumulation, and ferroptosis-related protein expression, other inflammatory stress or cell death programs, including apoptosis, pyroptosis, and senescence, may coexist in the nonunion microenvironment [59,60,61]. Third, the IL-1β-treated fracture model captures one inflammatory component of impaired repair but cannot fully reproduce the biomechanical complexity, chronicity, and comorbidity spectrum of human atrophic nonunion. Finally, the MR analysis was exploratory and depended on available GWAS instruments and outcome definitions. Future studies using genetic models, lineage tracing, chromatin-binding assays, and human nonunion specimens will be important to further test the IL-1β/EPAS1/ferroptosis axis in disease-relevant settings. Therefore, the IL-1β/EPAS1-associated ferroptotic program should be regarded as an important mechanism contributing to fracture nonunion, rather than the sole cause of atrophic fracture nonunion.

## 5. Conclusions

This study supports a model in which inflammatory IL-1β signaling is associated with an EPAS1-related ferroptotic program in SSPCs, contributing to impaired osteochondrogenic differentiation and defective fracture repair. Pharmacological EPAS1 inhibition attenuated IL-1β-induced ferroptotic stress in primary SSPCs and improved IL-1β-impaired bone regeneration in vivo. Together, these findings suggest that the IL-1β/EPAS1-associated ferroptotic program is an important mechanism contributing to SSPC dysfunction in inflammation-associated fracture nonunion, rather than the sole cause of atrophic nonunion. This pathway may represent a potential therapeutic target, warranting further validation in genetic models and clinically relevant nonunion settings.

## Data Availability

The public scRNA-seq dataset analyzed in this study is available from the Gene Expression Omnibus under accession number GSE242836. The in-house scRNA-seq dataset generated or analyzed in this study will be deposited in a public repository before publication; accession information will be provided during review. GWAS summary statistics used for Mendelian randomization were obtained from OpenGWAS and FinnGen, and the corresponding dataset IDs are provided in the Supplementary Materials. Other data supporting the findings of this study are available from the corresponding authors upon reasonable request.

## Supplementary Materials

The following supporting information can be downloaded at: https://www.mdpi.com/article/10.3390/cimb48060606/s1.

## Abbreviations

The following abbreviations are used in this manuscript:

SSPCs	Skeletal stem/progenitor cells
IIFCs	Injury-induced fibrogenic cells
NonU	Fracture nonunion
Frac	Normal fracture healing
PO	Periosteum
scRNA-seq	Single-cell RNA sequencing
UMAP	Uniform manifold approximation and projection
GO	Gene Ontology
KEGG	Kyoto Encyclopedia of Genes and Genomes
MR	Mendelian randomization
IVW	Inverse-variance weighted
SNP	Single-nucleotide polymorphism
LD	Linkage disequilibrium
qRT-PCR	Quantitative real-time PCR
CFU	Colony-forming unit
micro-CT	Micro-computed tomography
BV	Bone volume
BV/TV	Bone volume fraction
Fe^2+^	Ferrous iron
IL-1β	interleukin-1β
EPAS1	endothelial PAS domain protein 1
NF-κB	Nuclear Factor kappa-light-chain-enhancer of activated B cells

## References

[1] Mills L.A., Aitken S.A., Simpson A.H.R.W. (2017). The Risk of Non-Union per Fracture: Current Myths and Revised Figures from a Population of over 4 Million Adults. Acta Orthop..

[2] Zura R., Xiong Z., Einhorn T., Watson J.T., Ostrum R.F., Prayson M.J., Della Rocca G.J., Mehta S., McKinley T., Wang Z. (2016). Epidemiology of Fracture Nonunion in 18 Human Bones. JAMA Surg..

[3] Rupp M., Biehl C., Budak M., Thormann U., Heiss C., Alt V. (2018). Diaphyseal Long Bone Nonunions-Types, Aetiology, Economics, and Treatment Recommendations. Int. Orthop..

[4] Megas P. (2005). Classification of Non-Union. Injury.

[5] Stewart S.K. (2019). Fracture Non-Union: A Review of Clinical Challenges and Future Research Needs. Malays. Orthop. J..

[6] Hadjiargyrou M., O’Keefe R.J. (2014). The Convergence of Fracture Repair and Stem Cells: Interplay of Genes, Aging, Environmental Factors and Disease. J. Bone Min. Res..

[7] Nicholson J.A., Makaram N., Simpson A., Keating J.F. (2021). Fracture Nonunion in Long Bones: A Literature Review of Risk Factors and Surgical Management. Injury.

[8] Claes L., Recknagel S., Ignatius A. (2012). Fracture Healing under Healthy and Inflammatory Conditions. Nat. Rev. Rheumatol..

[9] Brown M.L., Yukata K., Farnsworth C.W., Chen D.-G., Awad H., Hilton M.J., O’Keefe R.J., Xing L., Mooney R.A., Zuscik M.J. (2014). Delayed Fracture Healing and Increased Callus Adiposity in a C57BL/6J Murine Model of Obesity-Associated Type 2 Diabetes Mellitus. PLoS ONE.

[10] Peel N.F., Moore D.J., Barrington N.A., Bax D.E., Eastell R. (1995). Risk of Vertebral Fracture and Relationship to Bone Mineral Density in Steroid Treated Rheumatoid Arthritis. Ann. Rheum. Dis..

[11] Wang C., Ying J., Nie X., Zhou T., Xiao D., Swarnkar G., Abu-Amer Y., Guan J., Shen J. (2021). Targeting Angiogenesis for Fracture Nonunion Treatment in Inflammatory Disease. Bone Res..

[12] Inzana J.A., Maher J.R., Takahata M., Schwarz E.M., Berger A.J., Awad H.A. (2013). Bone Fragility beyond Strength and Mineral Density: Raman Spectroscopy Predicts Femoral Fracture Toughness in a Murine Model of Rheumatoid Arthritis. J. Biomech..

[13] Xiao D., Fang L., Liu Z., He Y., Ying J., Qin H., Lu A., Shi M., Li T., Zhang B. (2023). DNA Methylation-Mediated Rbpjk Suppression Protects against Fracture Nonunion Caused by Systemic Inflammation. J. Clin. Investig..

[14] Manara M., Sinigaglia L. (2015). Bone and TNF in Rheumatoid Arthritis: Clinical Implications. RMD Open.

[15] Lawrence T. (2009). The Nuclear Factor NF-kappaB Pathway in Inflammation. Cold Spring Harb. Perspect. Biol..

[16] Liu J., Qiu C., Wang X., Xiang Z., Sun J., Chang M., Ma Q., Zhuang Y., Zhao Y., Yang Q. (2025). Sulfasalazine Disrupts the Interaction between TNFα and TNFR1 Thus Inhibiting NF-kB Signaling Activation to Promote Bone Fracture Healing. J. Pharmacol. Sci..

[17] Kopf M., Bachmann M.F., Marsland B.J. (2010). Averting Inflammation by Targeting the Cytokine Environment. Nat. Rev. Drug Discov..

[18] Osipov B., Paralkar M.P., Emami A.J., Cunningham H.C., Tjandra P.M., Pathak S., Langer H.T., Baar K., Christiansen B.A. (2022). Sex Differences in Systemic Bone and Muscle Loss Following Femur Fracture in Mice. J. Orthop. Res..

[19] Liu Y.L., Tang X.T., Shu H.S., Zou W., Zhou B.O. (2024). Fibrous Periosteum Repairs Bone Fracture and Maintains the Healed Bone throughout Mouse Adulthood. Dev. Cell.

[20] Xing W., Feng H., Jiang B., Gao B., Liu J., Xie Z., Zhang Y., Hu X., Sun J., Greenblatt M.B. (2024). Itm2a Expression Marks Periosteal Skeletal Stem Cells That Contribute to Bone Fracture Healing. J. Clin. Investig..

[21] Perrin S., Ethel M., Bretegnier V., Goachet C., Wotawa C.-A., Luka M., Coulpier F., Masson C., Ménager M., Colnot C. (2024). Single-Nucleus Transcriptomics Reveal the Differentiation Trajectories of Periosteal Skeletal/Stem Progenitor Cells in Bone Regeneration. eLife.

[22] Hachemi Y., Perrin S., Ethel M., Julien A., Vettese J., Geisler B., Göritz C., Colnot C. (2024). Multimodal Analyses of Immune Cells during Bone Repair Identify Macrophages as a Therapeutic Target in Musculoskeletal Trauma. Bone Res..

[23] Wang Z., Yan Q., Wang Z., Hu Z., Wang C., Zhang X., Gao X., Bai X., Chen X., Zhang L. (2024). Ferroptosis and Its Implications in Bone-Related Diseases. PeerJ.

[24] Li J., Cao F., Yin H.-L., Huang Z.-J., Lin Z.-T., Mao N., Sun B., Wang G. (2020). Ferroptosis: Past, Present and Future. Cell Death Dis..

[25] Sun X., Huang N., Li P., Dong X., Yang J., Zhang X., Zong W.-X., Gao S., Xin H. (2023). TRIM21 Ubiquitylates GPX4 and Promotes Ferroptosis to Aggravate Ischemia/Reperfusion-Induced Acute Kidney Injury. Life Sci..

[26] Chen S., Fu J., Long J., Liu C., Ai X., Long D., Leng X., Zhang Y., Liao Z., Li C. (2025). Bulk RNA-Seq Conjoined with ScRNA-Seq Analysis Reveals the Molecular Characteristics of Nucleus Pulposus Cell Ferroptosis in Rat Aging Intervertebral Discs. Arthritis Res. Ther..

[27] Xiang W., Zhang T., Li B., Li S., Zhang B., Fang S., Chen L., Gong Y., Huang B., Feng D. (2025). Senescent Macrophages Induce Ferroptosis in Skeletal Muscle and Accelerate Osteoarthritis-Related Muscle Atrophy. Nat. Aging.

[28] Wang W., Cheng Z., Yu M., Liu K., Duan H., Zhang Y., Huang X., Li M., Li C., Hu Y. (2025). Injectable ECM-Mimetic Dynamic Hydrogels Abolish Ferroptosis-Induced Post-Discectomy Herniation through Delivering Nucleus Pulposus Progenitor Cell-Derived Exosomes. Nat. Commun..

[29] de Seny D., Cobraiville G., Leprince P., Fillet M., Collin C., Mathieu M., Hauzeur J.-P., Gangji V., Malaise M.G. (2016). Biomarkers of Inflammation and Innate Immunity in Atrophic Nonunion Fracture. J. Transl. Med..

[30] Cui Y.C., Qiu Y.S., Wu Q., Bu G., Teh S.W., He G.Z., Mok P.L., Samrot A.V., Mariappan R., Higuchi A. (2020). Hypoxic-Mediated Oxidative Stress Condition and Hydroxyapatite-Inducing Osteogenic Differentiation of Human Mesenchymal Stem Cells: A Mathematical Modeling Study. J. Biomed. Nanotechnol..

[31] Ryu J.-H., Shin Y., Huh Y.H., Yang S., Chun C.-H., Chun J.-S. (2012). Hypoxia-Inducible Factor-2α Regulates Fas-Mediated Chondrocyte Apoptosis during Osteoarthritic Cartilage Destruction. Cell Death Differ..

[32] Hwang H.S., Park S.J., Lee M.H., Kim H.A. (2017). MicroRNA-365 Regulates IL-1β-Induced Catabolic Factor Expression by Targeting HIF-2α in Primary Chondrocytes. Sci. Rep..

[33] Zhang X., Hou L., Guo Z., Wang G., Xu J., Zheng Z., Sun K., Guo F. (2023). Lipid Peroxidation in Osteoarthritis: Focusing on 4-Hydroxynonenal, Malondialdehyde, and Ferroptosis. Cell Death Discov..

[34] Zhou X., Zheng Y., Sun W., Zhang Z., Liu J., Yang W., Yuan W., Yi Y., Wang J., Liu J. (2021). D-Mannose Alleviates Osteoarthritis Progression by Inhibiting Chondrocyte Ferroptosis in a HIF-2α-Dependent Manner. Cell Prolif..

[35] Singhal R., Mitta S.R., Das N.K., Kerk S.A., Sajjakulnukit P., Solanki S., Andren A., Kumar R., Olive K.P., Banerjee R. (2021). HIF-2α Activation Potentiates Oxidative Cell Death in Colorectal Cancers by Increasing Cellular Iron. J. Clin. Investig..

[36] Hao Y., Stuart T., Kowalski M.H., Choudhary S., Hoffman P., Hartman A., Srivastava A., Molla G., Madad S., Fernandez-Granda C. (2024). Dictionary Learning for Integrative, Multimodal and Scalable Single-Cell Analysis. Nat. Biotechnol..

[37] Korsunsky I., Millard N., Fan J., Slowikowski K., Zhang F., Wei K., Baglaenko Y., Brenner M., Loh P.-R., Raychaudhuri S. (2019). Fast, Sensitive and Accurate Integration of Single-Cell Data with Harmony. Nat. Methods.

[38] Zhou N., Yuan X., Du Q., Zhang Z., Shi X., Bao J., Ning Y., Peng L. (2023). FerrDb V2: Update of the Manually Curated Database of Ferroptosis Regulators and Ferroptosis-Disease Associations. Nucleic Acids Res..

[39] Yu G., Wang L.-G., Han Y., He Q.-Y. (2012). clusterProfiler: An R Package for Comparing Biological Themes among Gene Clusters. OMICS.

[40] Schubert M., Klinger B., Klünemann M., Sieber A., Uhlitz F., Sauer S., Garnett M.J., Blüthgen N., Saez-Rodriguez J. (2018). Perturbation-Response Genes Reveal Signaling Footprints in Cancer Gene Expression. Nat. Commun..

[41] Street K., Risso D., Fletcher R.B., Das D., Ngai J., Yosef N., Purdom E., Dudoit S. (2018). Slingshot: Cell Lineage and Pseudotime Inference for Single-Cell Transcriptomics. BMC Genom..

[42] Aibar S., González-Blas C.B., Moerman T., Huynh-Thu V.A., Imrichova H., Hulselmans G., Rambow F., Marine J.-C., Geurts P., Aerts J. (2017). SCENIC: Single-Cell Regulatory Network Inference and Clustering. Nat. Methods.

[43] Browaeys R., Saelens W., Saeys Y. (2020). NicheNet: Modeling Intercellular Communication by Linking Ligands to Target Genes. Nat. Methods.

[44] Skrivankova V.W., Richmond R.C., Woolf B.A.R., Yarmolinsky J., Davies N.M., Swanson S.A., VanderWeele T.J., Higgins J.P.T., Timpson N.J., Dimou N. (2021). Strengthening the Reporting of Observational Studies in Epidemiology Using Mendelian Randomization: The STROBE-MR Statement. JAMA.

[45] Wang Z., Wu Z., Wang H., Feng R., Wang G., Li M., Wang S.-Y., Chen X., Su Y., Wang J. (2023). An Immune Cell Atlas Reveals the Dynamics of Human Macrophage Specification during Prenatal Development. Cell.

[46] Kalucka J., de Rooij L.P.M.H., Goveia J., Rohlenova K., Dumas S.J., Meta E., Conchinha N.V., Taverna F., Teuwen L.-A., Veys K. (2020). Single-Cell Transcriptome Atlas of Murine Endothelial Cells. Cell.

[47] Gao Y., Li J., Cheng W., Diao T., Liu H., Bo Y., Liu C., Zhou W., Chen M., Zhang Y. (2024). Cross-Tissue Human Fibroblast Atlas Reveals Myofibroblast Subtypes with Distinct Roles in Immune Modulation. Cancer Cell.

[48] Chen Y., Fang Z.-M., Yi X., Wei X., Jiang D.-S. (2023). The Interaction between Ferroptosis and Inflammatory Signaling Pathways. Cell Death Dis..

[49] Zhang S., Xu J., Si H., Wu Y., Zhou S., Shen B. (2022). The Role Played by Ferroptosis in Osteoarthritis: Evidence Based on Iron Dyshomeostasis and Lipid Peroxidation. Antioxidants.

[50] Gao L., Hua W., Tian L., Zhou X., Wang D., Yang Y., Ni G. (2022). Molecular Mechanism of Ferroptosis in Orthopedic Diseases. Cells.

[51] Liu J., Zhou H., Chen J., Zuo Q., Liu F. (2024). Baicalin Inhibits IL-1β-Induced Ferroptosis in Human Osteoarthritis Chondrocytes by Activating Nrf-2 Signaling Pathway. J. Orthop. Surg. Res..

[52] Yao X., Sun K., Yu S., Luo J., Guo J., Lin J., Wang G., Guo Z., Ye Y., Guo F. (2021). Chondrocyte Ferroptosis Contribute to the Progression of Osteoarthritis. J. Orthop. Transl..

[53] Saito T., Fukai A., Mabuchi A., Ikeda T., Yano F., Ohba S., Nishida N., Akune T., Yoshimura N., Nakagawa T. (2010). Transcriptional Regulation of Endochondral Ossification by HIF-2alpha during Skeletal Growth and Osteoarthritis Development. Nat. Med..

[54] Yang S., Kim J., Ryu J.-H., Oh H., Chun C.-H., Kim B.J., Min B.H., Chun J.-S. (2010). Hypoxia-Inducible Factor-2alpha Is a Catabolic Regulator of Osteoarthritic Cartilage Destruction. Nat. Med..

[55] Wallace E.M., Rizzi J.P., Han G., Wehn P.M., Cao Z., Du X., Cheng T., Czerwinski R.M., Dixon D.D., Goggin B.S. (2016). A Small-Molecule Antagonist of HIF2α Is Efficacious in Preclinical Models of Renal Cell Carcinoma. Cancer Res..

[56] Courtney K.D., Infante J.R., Lam E.T., Figlin R.A., Rini B.I., Brugarolas J., Zojwalla N.J., Lowe A.M., Wang K., Wallace E.M. (2018). Phase I Dose-Escalation Trial of PT2385, a First-in-Class Hypoxia-Inducible Factor-2α Antagonist in Patients with Previously Treated Advanced Clear Cell Renal Cell Carcinoma. J. Clin. Oncol..

[57] Bowers K.M., Anderson D.E. (2024). Delayed Union and Nonunion: Current Concepts, Prevention, and Correction: A Review. Bioengineering.

[58] Gómez-Barrena E., Ehrnthaller C. (2024). Long Bone Uninfected Non-Union: Grafting Techniques. EFORT Open Rev..

[59] Yang J., Hu S., Bian Y., Yao J., Wang D., Liu X., Guo Z., Zhang S., Peng L. (2022). Targeting Cell Death: Pyroptosis, Ferroptosis, Apoptosis and Necroptosis in Osteoarthritis. Front. Cell Dev. Biol..

[60] Wu Q., Du J., Bae E.J., Choi Y. (2024). Pyroptosis in Skeleton Diseases: A Potential Therapeutic Target Based on Inflammatory Cell Death. Int. J. Mol. Sci..

[61] Lawrence M., Goyal A., Pathak S., Ganguly P. (2024). Cellular Senescence and Inflammaging in the Bone: Pathways, Genetics, Anti-Aging Strategies and Interventions. Int. J. Mol. Sci..

