# Supplementary material for: IL-1β/EPAS1-Associated Ferroptotic Stress Impairs Skeletal Stem/Progenitor Cell Function in Inflammation-Associated Fracture Nonunion
Source: Curr Issues Mol Biol. 2026 Jun 9;48(6):606. doi: 10.3390/cimb48060606 (PMC13298421; doi:10.3390/cimb48060606)
Supplement: Supplementary file 1 [file cimb-48-00606-s001.zip › cimb-4330778-supplementary.pdf]

Article

IL-1β/EPAS1-Associated Ferroptotic Stress Impairs Skeletal Stem/Progenitor Cell Function in Inflammation-Associated Fracture Nonunion

Supplementary Figures

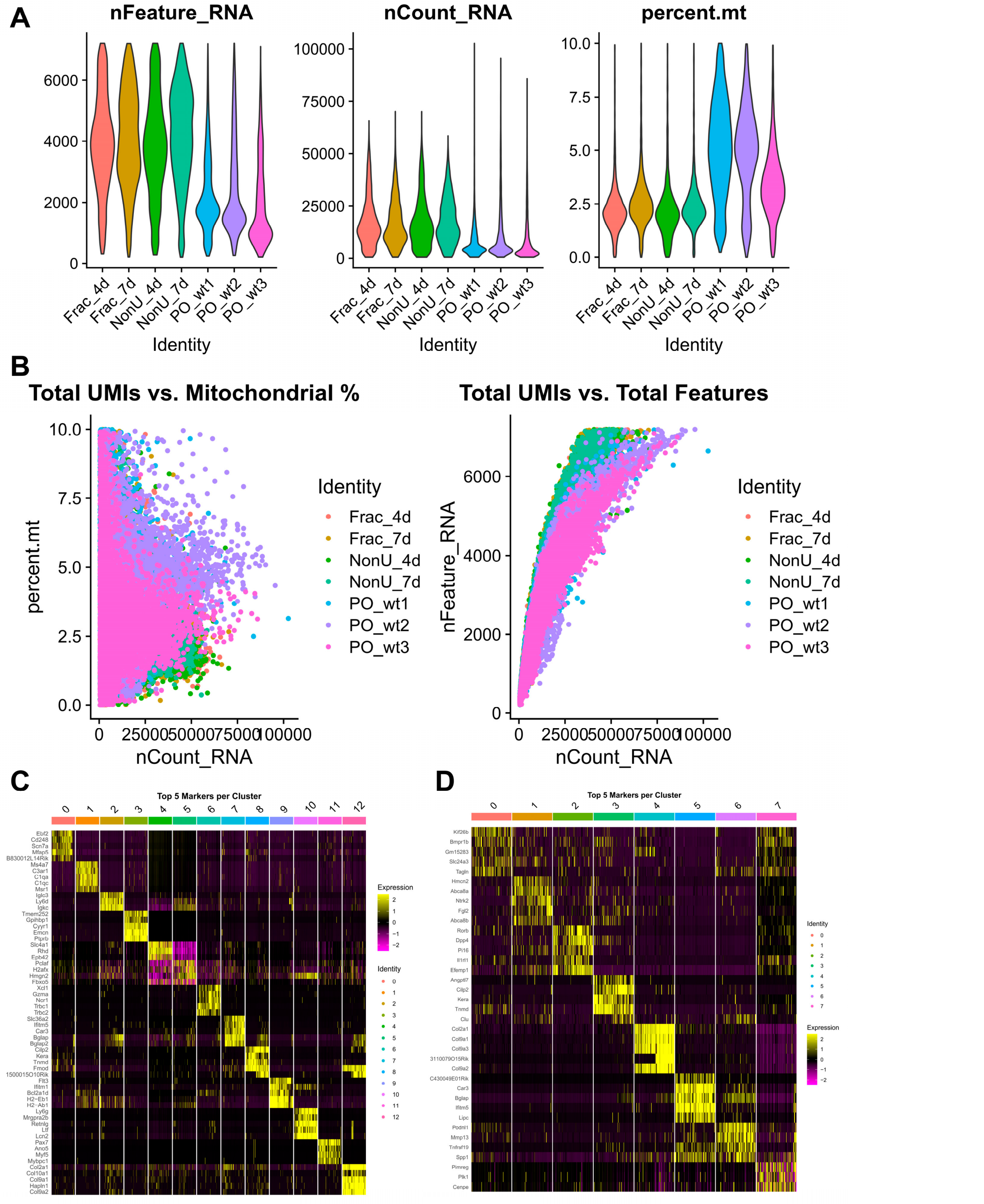

Figure S1. Quality control and marker-based annotation of the scRNA-seq. **(A)** Violin plots showing the distribution of detected genes (nFeature_RNA), total transcript counts (nCount_RNA), and mitochondrial gene percentage (percent.mt) across samples after filtering. **(B)** Scatter plots showing the relationships between nCount_RNA and percent.mt, and between nCount_RNA and nFeature_RNA, used for quality control assessment. **(C)** Heatmap showing the top five marker genes for the 12 annotated major cell populations, including stromal, immune, and vascular compartments.

(D) Heatmap showing the top five marker genes for the eight stromal cell subsets identified by sub-clustering analysis.

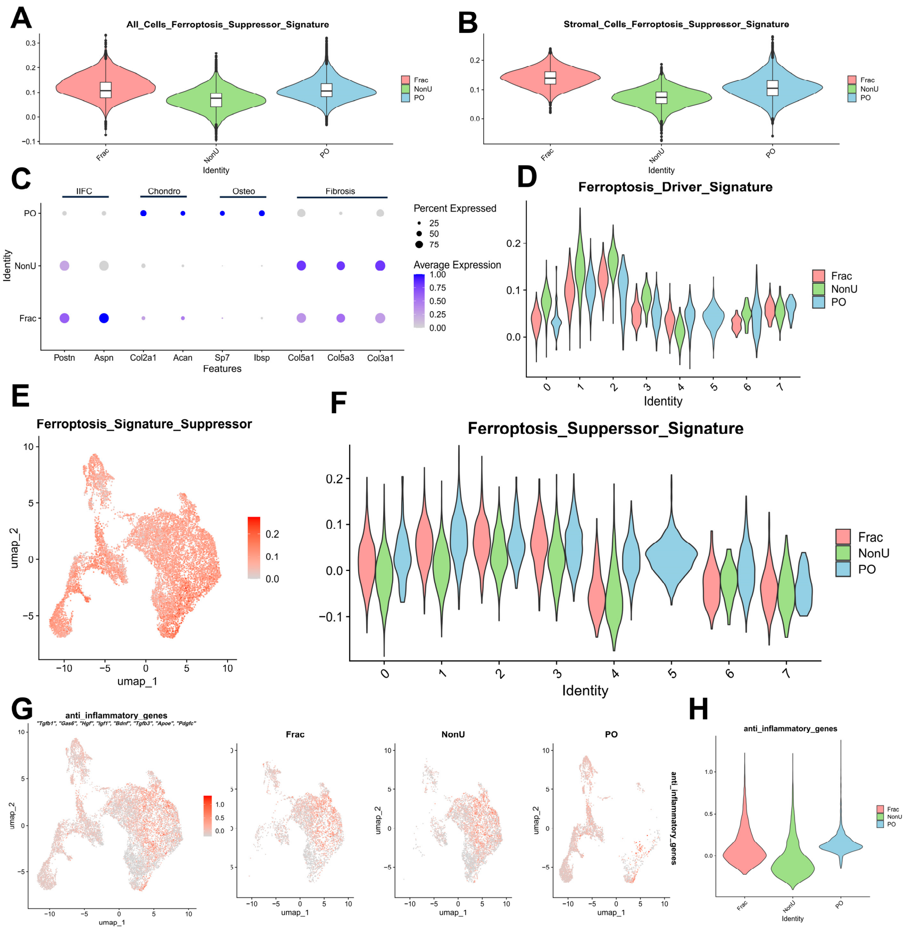

Figure S2. Supplementary analysis of ferroptosis and inflammatory signatures in stromal subsets. **(A)** Violin plots showing the ferroptosis suppressor signature score in all cells, split by condition. **(B)** Violin plots showing the ferroptosis suppressor signature score in the stromal cell compartment, split by condition. **(C)** Dot plot showing the expression of representative chondrogenic (*Col2a1*, *Acan*), osteogenic (*Bglap*, *Sp7*), and fibrotic (*Col3a1*, *Postn*) genes within IIFCs across the three conditions. **(D)** Violin plots of the ferroptosis driver score across the 8 stromal subsets, split by condition and group. **(E)** Feature plots of the ferroptosis suppressor score on the stromal UMAP, split by condition. **(F)** Violin plots of the ferroptosis suppressor score across the 8 stromal subsets, split by condition. **(G)** Feature plots of the anti-inflammatory score on the stromal UMAP, split by condition. **(H)** Violin plots of the anti-inflammatory score across the 8 stromal subsets, split by condition.

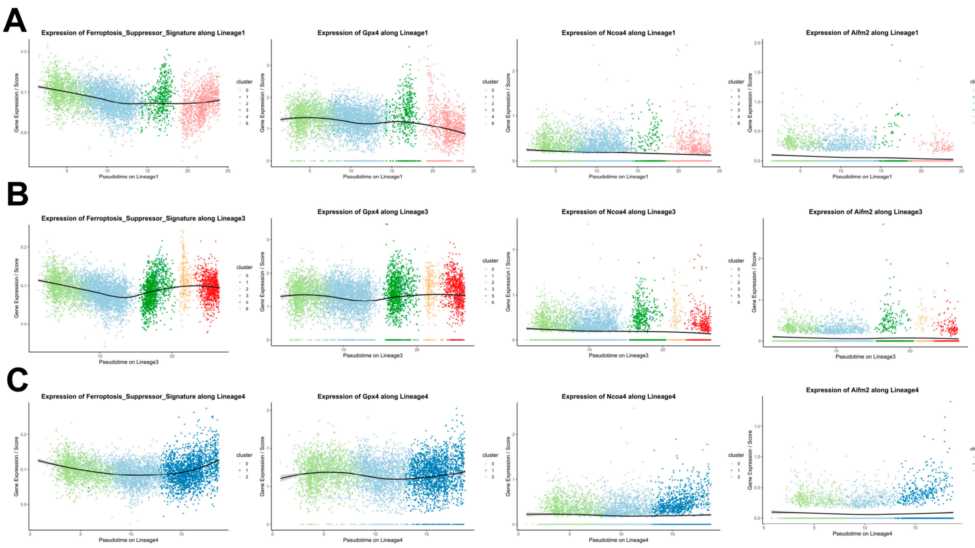

Figure S3. Ferroptosis-suppressor gene expression along stromal pseudotime trajectories. **(A)** Scatter plots showing the expression of the ferroptosis suppressor signature, *Gpx4*, *Ncoa4*, and *Aifm2* (from left to right) along the normal chondrogenic (Lineage 1) trajectory over pseudotime. **(B)** Scatter plots showing the expression of the ferroptosis suppressor signature, *Gpx4*, *Ncoa4*, and *Aifm2* (from left to right) along the normal osteogenic (Lineage 3) trajectory over pseudotime. **(C)** Scatter plots showing the expression of the ferroptosis suppressor signature, *Gpx4*, *Ncoa4*, and *Aifm2* (from left to right) along the pathological (Lineage 4) trajectory over pseudotime.

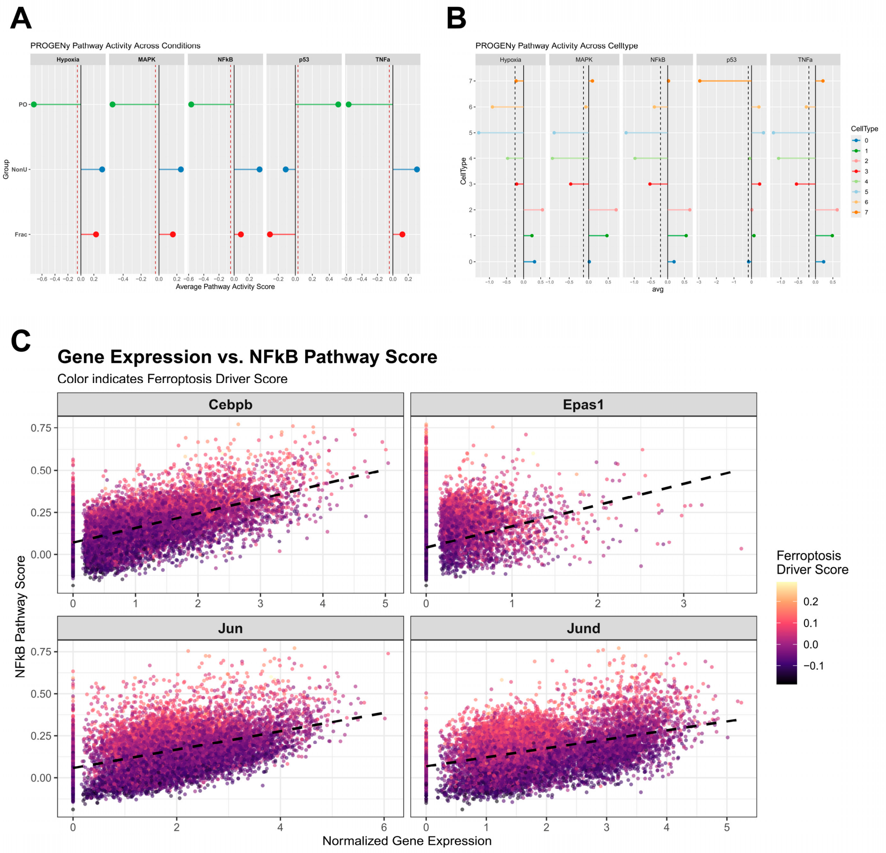

Figure S4. Pathway activity analysis supports activation of inflammatory signaling in nonunion-associated stromal cells. **(A)** Bar plot showing PROGENy-inferred pathway activity scores averaged across stromal cells and split by experimental condition. The dashed line indicates the average pathway activity score. **(B)** Bar plot showing PROGENy-inferred pathway activity scores across stromal

subsets in the NonU group, including NF-κB, MAPK, and TNF-α pathway activities in SSPCs and IIFC subsets. **(C)** Scatter plot showing the association between NF-κB pathway activity and the combined regulon activity of candidate transcription factors, including Epas1, Jun, Jund, and Cebpb. Each dot represents one cell and is colored by ferroptosis-driver module score. The dashed line indicates the linear.

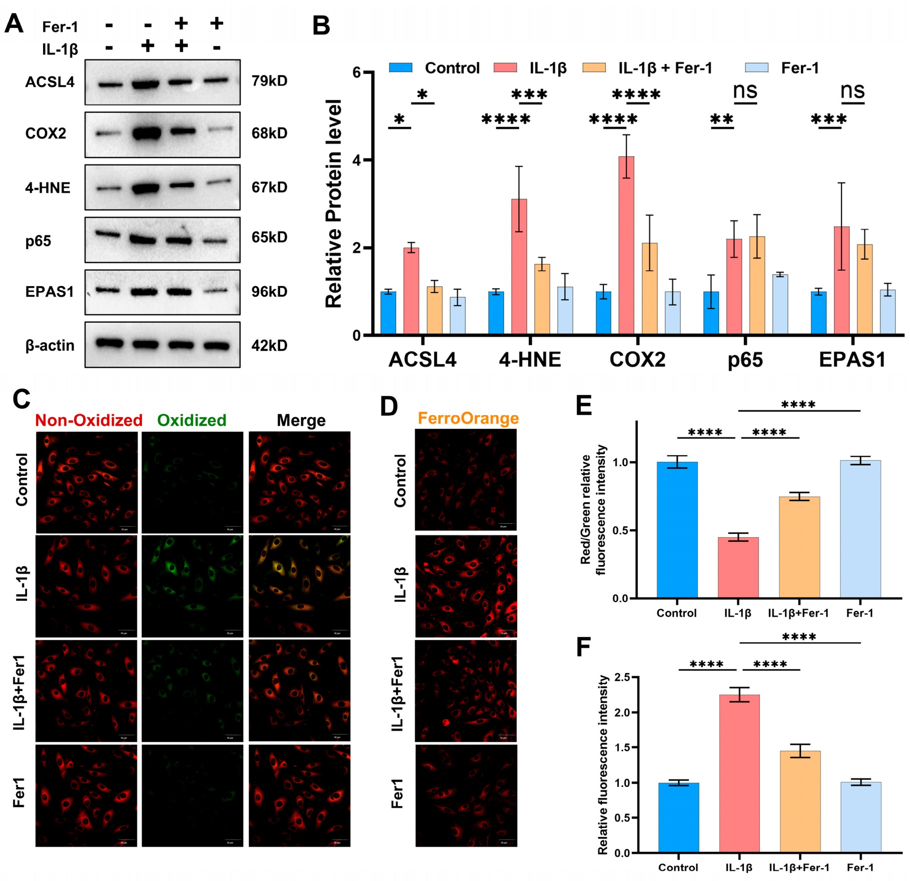

Figure S5. Ferrostatin-1 attenuates IL-1β-induced lipid peroxidation, Fe^{2+} accumulation, and ferroptosis-related molecular changes in SSPCs.

(A) Representative western blot images showing ACSL4, 4-HNE, COX2, p65, and EPAS1 expression in SSPCs treated with vehicle control, IL-1β, IL-1β plus Fer-1, or Fer-1 alone. β-actin was used as the loading control; **(B)** Quantification of western blot signals shown in (A). IL-1β increased ACSL4, 4-HNE, COX2, p65, and EPAS1 levels. Fer-1 co-treatment attenuated IL-1β-induced ACSL4, 4-HNE, and COX2 upregulation, whereas p65 and EPAS1 were not significantly reduced by Fer-1 co-treatment; **(C)** Representative C11-BODIPY fluorescence images showing lipid peroxidation under different treatment conditions; **(D)** Representative FerroOrange fluorescence images showing intracellular Fe^{2+} accumulation under different treatment conditions; **(E)** Quantification of the C11-BODIPY red/green fluorescence intensity ratio. A decreased red/green ratio indicates increased lipid peroxidation; **(F)** Quantification of FerroOrange fluorescence intensity. Increased fluorescence intensity reflects intracellular Fe^{2+} accumulation. Data are presented as mean ± SD. Statistical significance was determined by one way ANOVA; **$P < 0.01$, ***$P < 0.001$, ****$P < 0.0001$; ns, not significant.

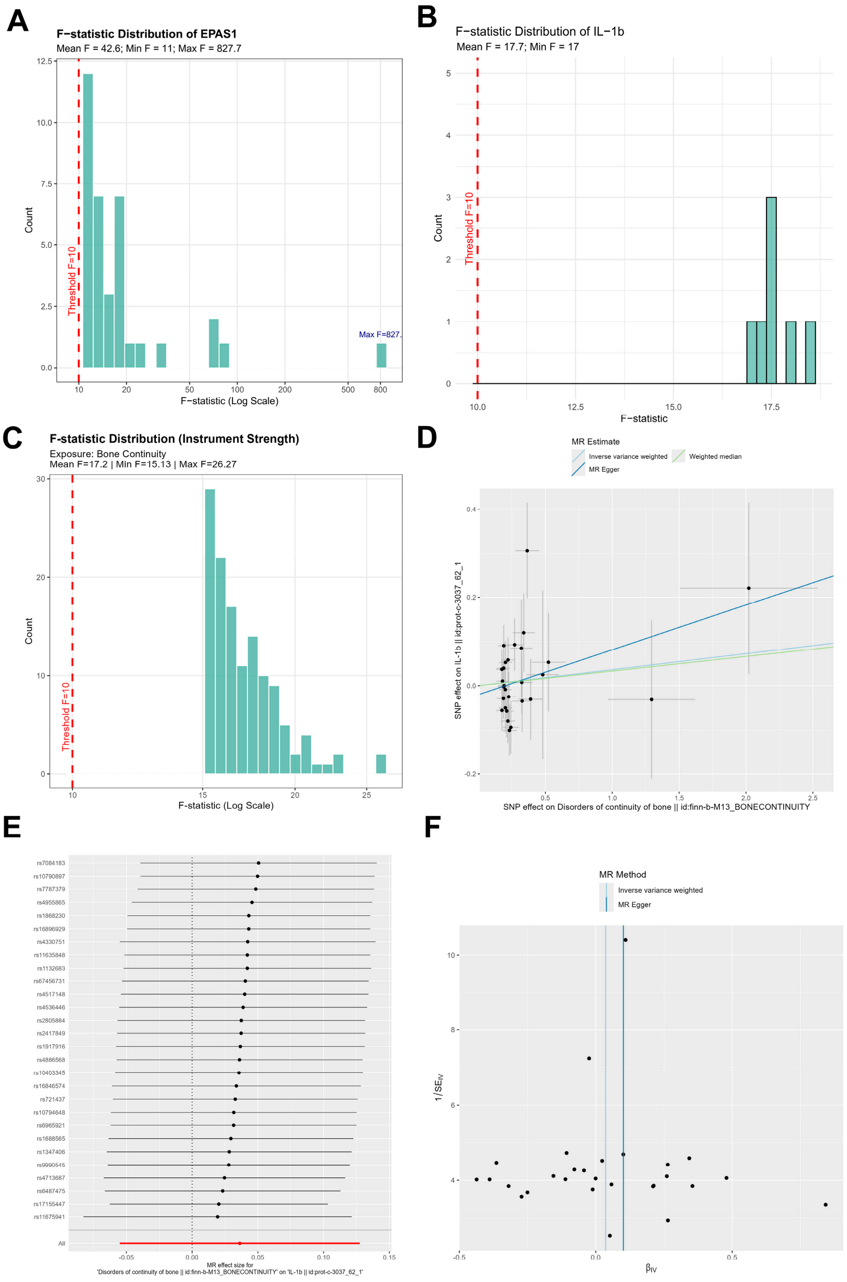

Figure S6. Instrument strength and reverse Mendelian randomization analysis. **(A)** Bar plot showing F-statistics for independent SNPs used as instrumental variables for *EPAS1* in the forward Mendelian randomization analysis. The dashed line indicates the conventional weak-instrument threshold of F = 10. **(B)** Bar plot showing F-statistics for instrumental variables associated with IL-1β in the forward Mendelian randomization analysis. **(C)** Bar plot showing F-statistics for bone nonunion-associated SNPs used as instrumental variables in the reverse Mendelian randomization analysis. Values greater than 10 indicate adequate instrument strength**. (D)** Scatter plot showing reverse Mendelian randomization analysis of genetically predicted bone nonunion liability on IL-1β. The slope represents the causal estimate. **(E)** Leave-one-out sensitivity analysis for the reverse association between bone nonunion liability and IL-1β. **(F)** Funnel plot assessing potential directional pleiotropy in the reverse Mendelian randomization analysis of bone nonunion liability on IL-1β. regression fit.
